# Quantum advantage in variational Bayes inference

**DOI:** 10.1073/pnas.2212660120

**Published:** 2023-07-25

**Authors:** Hideyuki Miyahara, Vwani Roychowdhury

**Affiliations:** ^a^Department of Electrical and Computer Engineering, Henry Samueli School of Engineering and Applied Science, University of California, Los Angeles, CA 90095

**Keywords:** quantum machine learning, variational Bayes inference, quantum annealing, deterministic annealing

## Abstract

Variational Bayes (VB) inference algorithm is used widely to estimate both the parameters and the unobserved hidden variables in generative statistical models. The algorithm—inspired by variational methods used in computational physics—is iterative and can get easily stuck in local minima, even when classical techniques, such as deterministic annealing (DA), are used. We study a VB inference algorithm based on a nontraditional quantum annealing approach—referred to as quantum annealing variational Bayes (QAVB) inference—and show that there is indeed a quantum advantage to QAVB over its classical counterparts. In particular, we show that such better performance is rooted in key quantum mechanics concepts: i) The ground state of the Hamiltonian of a quantum system—defined from the given data—corresponds to an optimal solution for the minimization problem of the variational free energy at very low temperatures; ii) such a ground state can be achieved by a technique paralleling the quantum annealing process; and iii) starting from this ground state, the optimal solution to the VB problem can be achieved by increasing the heat bath temperature to unity, and thereby avoiding local minima introduced by spontaneous symmetry breaking observed in classical physics based VB algorithms. We also show that the update equations of QAVB can be potentially implemented using ⌈log*K*⌉ qubits and 𝒪(*K*) operations per step, where *K* is the number of values hidden categorical variables can take. Thus, QAVB can match the time complexity of existing VB algorithms, while delivering higher performance.

## Significance

Quantum machine learning (QML) is an emerging research field that deals with quantum algorithms for data analysis. It is hoped that QML will yield practical demonstrations of quantum advantage by exploiting the emerging noisy intermediate-scale quantum (NISQ) devices, which cannot yet implement large-scale quantum algorithms. Most of the proposed QML frameworks, such as quantum principal component analysis, quantum circuits, and quantum recommendation systems, provide potential quantum speedups of corresponding classical algorithms. These algorithms, thus, do not improve the quality of the solutions, and QML algorithms that outperform classical ML schemes are rare or nonexistent. This paper shows an example of how quantum mechanics can lead to better solutions for machine learning (ML) problems without incurring increased time complexity overheads.

Quantum machine learning (QML) primarily deals with quantum algorithms and quantum-inspired algorithms for data analysis and is an emerging research field that is forming new bridges between the traditional fields of physics and machine learning. Several QML frameworks, such as quantum principal component analysis (qPCA) (1) and quantum recommendation systems (2), have been introduced that show significant quantum speedups while achieving the same performance as the corresponding classical algorithms. These quantum algorithms, in turn, were later shown to have classical counterparts, and randomized algorithms with the same time complexity were derived (3, 4). This discovery process showed an encouraging synergy where the principles of quantum mechanics can also facilitate the design of better classical algorithms. Interest in QML has also been fueled by the emergence of noisy intermediate-scale quantum (NISQ) devices. The low fidelity and limited scale of such devices prevent implementations of well-known algorithms such as the Shor’s factorization algorithm or combinatorial optimization algorithms based on quantum annealing. However, efficient QML algorithms for conventional machine learning (ML) tasks, such as dimensionality reduction, clustering, classification, and Bayesian inference, could likely be implemented on NISQ devices and show potential quantum advantages in speed or accuracy. For example, variational quantum classifiers (VQC) and quantum circuit learning (QCL) frameworks have been proposed that hold the promise of time and hardware efficient training and realizations of conventional classifiers (5–7). Recent results, however, show that simple kernel method-based classical classifiers are guaranteed to have better performance than their quantum counterparts. Furthermore, there is no analytical guarantee or numerical evidence suggesting that the variational quantum algorithms will even have reasonable performance, especially for high-dimensional datasets where these algorithms are expected to have speedup advantages.

The above-mentioned examples underscore the general trend in the QML field: Existing algorithms can potentially speed up classical algorithms, but QML algorithms that outperform their classical ML counterparts are very rare or nonexistent. Thus, the search for QML algorithms that either perform better than any classical algorithm without incurring significant computational overheads or exhibit significant speedups (for the same performance) continues to remain an active area of interest. More interestingly, there is a report of utilizing NISQ devices for ML (8).

In this paper, we address the problem of variational Bayes (VB) inference, which is a popular technique in ML, and explore how quantum mechanics can help design an algorithm with better performance than the existing classical techniques. In fact, principles from classical statistical physics have already inspired a genre of algorithms for VB. The history of optimization algorithms motivated by physics dates back to simulated annealing (SA) (9), which utilizes a thermostat to overcome the local optima problem in optimization, and SA has been applied to several ML tasks (10, 11). SA approaches, however, have a well-known drawback in that they require an infinitely long annealing schedule to guarantee the global optimum of an optimization problem or at least a very long annealing schedule to reach its equilibrium state at a finite temperature. To fix such drawbacks of SA, deterministic annealing (DA) was developed and applied to several machine learning problems (12). For example, by applying DA to variational Bayes (VB) inference (13, 14), deterministic annealing variational Bayes (DAVB) inference (15) was proposed. However, it has been shown that DA and DAVB can get stuck in a local optimum relatively easily or a saddle point (as shown in Fig. 2*C*), and details of this phenomenon are further discussed later in this paper.

More recently, by following the trend in QML, a quantum annealing variational Bayes (QAVB) inference framework—a quantum-mechanical extension of VB and DAVB—was proposed in ref. 16, and the study showed that QAVB outperformed both VB and DAVB in several numerical examples. Other than numerical examples, concrete mechanisms that enable QAVB to achieve better performance than its classical counterparts were not given, and numerical results providing evidence for such potential mechanisms were not presented. Moreover, if QAVB is implemented classically, then each iteration step requires 𝒪(*K*^3^) operations (where the categorical hidden variable in the VB problem has *K* possible values), as compared to 𝒪(*K*) computations required by classical VB algorithms. Thus, any performance enhancements offered by QAVB seem to have an associated computational price. This increased computational cost stems from classical simulations of a quantum system, which requires repeated diagonalization of the underlying Hamiltonian. Thus, a natural question, especially in the context of QML, is whether the update equations of QAVB can be simulated using quantum devices, where no such diagonalization would be necessary.

In this paper, we first introduce the VB problem. Then, we explain the motivation behind the incorporation of a nontraditional quantum annealing (QA) approach and the framework of QAVB and formulate a mechanism by which QAVB could show better performance than both VB and DAVB. As in the traditional QA case, our nontraditional QA considers the evolution of a quantum system under a time-varying Hamiltonian; however, the evolution dynamics is now driven by relaxation under the mean-field (MF) approximation, as opposed to relaxation under the Schrödinger equation. We provide both numerical evidence and analytical proofs supporting this mechanism. From an analytical perspective, we show how the ground-state dynamics introduced by our nontraditional QA can also be analyzed by techniques similar to those used in the well-known adiabatic theorem that characterizes the traditional QA process where the Hamiltonian is time varying. In order to support our theoretical and mechanism-related results, we provide numerical results on two synthetic datasets, created using a generative model, where all the hidden variables and parameters are specified. This allows us to compare the performance of any algorithm to that of the ground-truth optimal solutions. As predicted, our results show that QAVB (a single run, independent of initial conditions) finds good estimates that are very close to that of the underlying generative models, but VB and DAVB find them with low probability. Moreover, these numerical results show that the QA part of QAVB is critically important for optimal parameter estimation and is the key to obtaining better performance than classical algorithms. For results on higher dimensional datasets where QAVB outperforms VB and DAVB, please refer to ref. 16. Then, we show that the QAVB update steps are completely positive and trace-preserving (CPTP) maps. Since it is known that a CPTP map can be implemented on quantum systems, we thus show that QAVB can be implemented using NISQ devices, comprising only ⌈log*K*⌉ qubits.

## Variational Bayes Inference

Suppose that we have *N* observable data points $Z^{obs}:={\left\{z_{i}^{obs}\right\}}_{i=1}^{N}$ that are the output of an unknown generative model $p_{gen}^{z}\left(z\right):\;z_{i}^{obs}∼p_{gen}^{z}\left(\cdot \right)$ with additional dynamics that are not necessarily observed. One of the important approaches in ML is to assume that the generative model can be well approximated by a parameterized model that outputs both the observable data points *Z*^obs^ as well as an associated set of unobservable or hidden data points, $\Sigma :={\left\{\sigma_{i}\right\}}_{i=1}^{N}$, where *σ*_*i*_ ∈ {1, 2, …, *K*} is a categorical variable with *K* outcomes. These hidden variables often have interpretable meanings and can be used to predict other outcomes associated with the dataset. The task then is to estimate the parameters of the generative model and the posterior distributions of the unobservable variables from the observable data.

More specifically, we first introduce the underlying model via a distribution *p*^*z*, *σ*|*θ*^(*z*, *σ*|*θ*), which is the conditional probability distribution of *z* and *σ* when *θ* is given, and $p_{\text{pr}}^{\theta }\left(\theta \right)$, which is the prior distribution of *θ*. Here, *θ* is the set of parameters that characterize the conditional distribution and $\Sigma :={\left\{\sigma_{i}\right\}}_{i=1}^{N}$ is the set of unobservable variables. For the above modeling to be successful, *p*^*z*, *σ*|*θ*^(*z*, *σ*|*θ*) and $p_{gen}^{z}\left(z\right)$ have to satisfy $p_{gen}^{z}\left(\cdot \right)\approx \sum_{S^{\sigma }}{}_{\sigma \in S^{\sigma }}p^{z,\sigma |\theta }\left(\cdot ,\sigma |\theta_{*}\right)$, where θ_*_ is an optimal parameter and *S*^*σ*^ is the domain of *σ*. For later convenience, we also define $p^{Z,\Sigma |\theta }\left(Z,\Sigma |\theta \right):=\prod_{i=1}^{N}p^{z,\sigma |\theta }\left(z_{i},\sigma_{i}|\theta \right)$.

Then, VB is an algorithm to compute the posterior distribution of *θ* and the hidden variables Σ in the above setup. In particular, the posterior distribution $p^{\Sigma ,\theta |Z}\left(\Sigma ,\theta |Z^{\mathrm{obs}}\right)\;:=\;\frac{p^{Z,\Sigma |\theta }\left(Z^{\mathrm{obs}},\Sigma |\theta \right)p_{\mathrm{pr}}^{\theta }\left(\theta \right)}{p^{Z}\left(Z^{\mathrm{obs}}\right)}$ is computationally intractable, as it is difficult to compute *p*^*Z*^(*Z*^obs^). Note that $p^{Z}\left(Z^{obs}\right):=\sum {}_{\Sigma \in S^{\Sigma }}\int_{\theta \in S^{\theta }}d\theta \;p^{Z,\Sigma |\theta }\left(Z^{obs},\Sigma |\theta \right)p_{\text{pr}}^{\theta }\left(\theta \right)$. Then, in VB, we try to approximate *p*^Σ,*θ*|*Z*^(Σ,*θ*|*Z*^obs^) by introducing a variational function *q*^Σ,*θ*^(Σ,*θ*) and minimizing the Kullback–Leibler (KL) divergence between *q*^Σ,*θ*^(Σ,*θ*) and *p*^Σ,*θ*|*Z*^(Σ,*θ*|*Z*^obs^). Specifically, we solve

[1] $$q_{*}^{\text{Σ},\theta }(\text{Σ},\theta )=\underset{q^{\text{Σ},\theta }(\text{Σ},\theta )}{\arg\;\min}\;\text{KL}\left(q^{\text{Σ},\theta }\left(\text{Σ},\theta \right)\left\|p^{\text{Σ},\theta |Z}\left(\text{Σ},\theta |Z^{\text{obs}}\right)\right.\right),$$

where the KL divergence between *p*(*x*) and *q*(*x*), defined over their domain *S*^*x*^, is given by

[2] $$\text{KL}\left(p\left(x\right)\|q\left(x\right)\right):=\sum_{x\in S^{x}}p\left(x\right)\left[\ln\;p\left(x\right)-\ln\;q\left(x\right)\right].$$

In Eq. **2**, *x* is assumed to be discrete, but almost the same definition is applicable for a continuous variable by replacing the summation with an integral. Furthermore, after making the assumption of MF where *q*^Σ,*θ*^(Σ,*θ*)=*q*^Σ^(Σ)*q*^*θ*^(*θ*), the optimization problem on the right-hand side of Eq. **1** is solved iteratively by setting

[3] $$\begin{array}{l} q_{t+1}^{\Sigma }\left(\Sigma \right)\\ \propto \exp\left(\int_{\theta \in S^{\theta }}d\theta \;q_{t+1}^{\theta }\ln\left(p^{Z,\Sigma |\theta }\left(Z^{obs},\Sigma |\theta \right)p_{\text{pr}}^{\theta }\left(\theta \right)\right)\right), \end{array}$$

[4] $$\begin{array}{l} q_{t+1}^{\theta }\left(\theta \right)\\ \propto \exp\left(\underset{\Sigma \in S^{\Sigma }}{\sum }q_{t}^{\Sigma }\left(\Sigma \right)\ln\left(p^{Z,\Sigma |\theta }\left(Z^{obs},\Sigma |\theta \right)p_{\text{pr}}^{\theta }\left(\theta \right)\right)\right) \end{array}$$

where $q_{t}^{\Sigma }\left(\Sigma \right)$ and $q_{t}^{\theta }\left(\theta \right)$ are the distributions of Σ and *θ* at the *t*-th iteration, respectively (13, 14). Once we get the posterior distributions of *θ* and Σ, we can utilize them for inference problems.

## Motivations of Quantization and a Noncommutative Term

The optimization problem in Eq. **1**, however, is still highly nonconvex with multiple local minima, and finding good solutions is a challenging task. We explain this difficulty of VB from the viewpoint of quantum statistical mechanics and then show how this seeming escalation of complexity introduced by viewing a Bayesian problem as a quantum system leads to a better solution to the original VB problem. In statistical physics, the probability *w*_*n*_(*β*)^*^ of finding a system in a configuration with energy *ε*_*n*_ is given by $w_{n}\left(\beta \right):=e^{-\beta \in_{n}}/Z\left(\beta \right)$, where *β* := (*k*_B_*T*)^−1^, *k*_B_ is the Boltzmann constant, *T* is the temperature of a heat bath to which the system is attached, and $Z\left(\beta \right):=\sum_{n=0}^{\infty }e^{-\beta \varepsilon_{n}}$. We can now reverse directions, and, given the VB problem, we can construct a virtual physical system such that *p*^*Z*, Σ|*θ*^(*Z*^obs^, Σ|*θ*) is the probability of it being in configuration {*Z*^obs^, Σ} conditioned by *θ*. Then, this system is defined by energy levels $\varepsilon_{\Sigma |\theta }=-\frac{1}{\beta }\ln p^{Z,\Sigma |\theta }\left(Z^{\mathrm{obs}},\Sigma |\theta \right)$. Since the next step is to construct a virtual quantum system, it is more convenient to use the concept of a Hamiltonian, which specifies the energy level corresponding to every configuration of a system; for our classical system, the Hamiltonian is identical to the energy levels. We first define two Hamiltonians corresponding to the probabilities, *p*^*Z*, Σ|*θ*^(*Z*^obs^, Σ|*θ*) and $p_{\text{pr}}^{\theta }\left(\theta \right)$:

[5] $$H_{\text{cl}}^{\text{Σ}|\theta }:=-\ln p^{Z,\text{Σ}|\theta }\left(Z^{\text{obs}},\text{Σ}|\theta \right),$$

[6] $$H_{\text{pr}}^{\theta }:=-\ln p_{\text{pr}}^{\theta }(\theta ).$$

Next, we convert this classical physical system to quantum ones using the canonical quantization approach (17). We denote the projection operator on Σ and *θ* by ${\hat{P}}^{\Sigma ,\theta }:=\left|\Sigma ,\theta \rangle \langle \Sigma ,\theta \right|$; then, we can write the Hamiltonian operators of Eqs. **5** and **6** as

[7] $${\hat{H}}_{\text{cl}}^{\Sigma |\theta }:=\underset{\Sigma \in S^{\Sigma }}{\sum }\int_{\theta \in S^{\theta }}d\theta \;H_{\text{cl}}^{\Sigma |\theta }{\hat{P}}^{\Sigma ,\theta },$$

[8] $${\hat{H}}_{\text{pr}}^{\theta }:=\sum_{\Sigma \in S^{\Sigma }}\int_{\theta \in S^{\theta }}d\theta \;H_{\text{pr}}^{\theta }{\hat{P}}^{\Sigma ,\theta },$$

where *S*^Σ^ and *S*^*θ*^ are the domains of Σ and *θ*, respectively. Note that the dimension of the Hamiltonian is *K*^*N*^ if *θ* is not quantized and infinity if *θ* is quantized. These Hamiltonians are still diagonal, and thus, the system is still classical. Each, diagonal element is by definition, *ε*_Σ|*θ*_ = −ln*p*^*Z*, Σ|*θ*^(*Z*^obs^, Σ|*θ*). Since we are soon going to develop the framework for estimating these probabilities by defining a nondiagonal Hamiltonian, it is useful to introduce the notation of the Gibbs operator

[9] $$\hat{f}\left(\beta_{\text{pr}},\beta \right):=\exp\left(-\beta_{\text{pr}}{\hat{H}}_{\text{pr}}^{\theta }-\beta {\hat{H}}_{\text{cl}}^{\Sigma |\theta }\right),$$

and rewrite the probabilities back in terms of the Hamiltonian notation. For simplicity, we consider the case of a noninformative prior distribution, so that ${\hat{H}}_{\mathrm{pr}}^{\theta }$ is not a function of *θ*. Since we are still dealing with a diagonal Hamiltonian, we can rewrite Eq. **1** in the Hamiltonian formulation:

[10] $${\hat{\rho }}_{*}^{\Sigma ,\theta }\left(\Sigma ,\theta \right)=\underset{{\hat{\rho }}^{\Sigma ,\theta }}{\arg\;\min}S{\left.\left({\hat{\rho }}^{\Sigma ,\theta }\left\|\frac{e^{-\beta {\hat{H}}_{\text{cl}}^{\Sigma |\theta }}}{Z\left(\beta \right)}\right.\right)\right|}_{\beta =1},$$

where 𝒵(*β*) is the partition function at *β*: $Z\left(\beta \right):=\mathrm{Tr}[e^{-\beta {\hat{H}}_{\mathrm{cl}}^{\Sigma |\theta }}]$ and *β* is the inverse temperature. Furthermore, 𝒮(⋅∥⋅) is the quantum relative entropy, which is a quantum extension of the KL divergence, Eq. **2**, given by

[11] $$S\left(\hat{\rho }||\hat{\sigma }\right):=\text{Tr}\left[\hat{\rho }\ln\hat{\rho }-\hat{\rho }\ln\hat{\sigma }\right].$$

The optimization problem in Eq. **10** is as difficult as Eq. **1** since they are equivalent.

Let us first consider a simpler problem by taking the limit *β* = ∞ in Eq. **10**; then, we have a

[12] $$|0;\text{cl}\rangle \langle 0;\text{cl}|=\underset{{\hat{\rho }}^{\Sigma ,\theta }}{\arg\;\min}\underset{\beta \to \infty }{\lim}S\left({\hat{\rho }}^{\Sigma ,\theta }\left\|\frac{e^{-\beta {\hat{H}}_{\text{cl}}^{\Sigma |\theta }}}{Z\left(\beta \right)}\right.\right),$$

where |0; cl⟩ is the ground state of ${\hat{H}}_{\mathrm{cl}}^{\Sigma |\theta }$. Eq. **12** implies that at *β* = ∞, Eq. **1** becomes the problem of finding the ground state of the data-defined Hamiltonian in Eq. **7**. As explained next, we can use a variant of the quantum annealing technique to approximate such a ground state, denoted as |0; cl⟩.

**Fig. 1.**
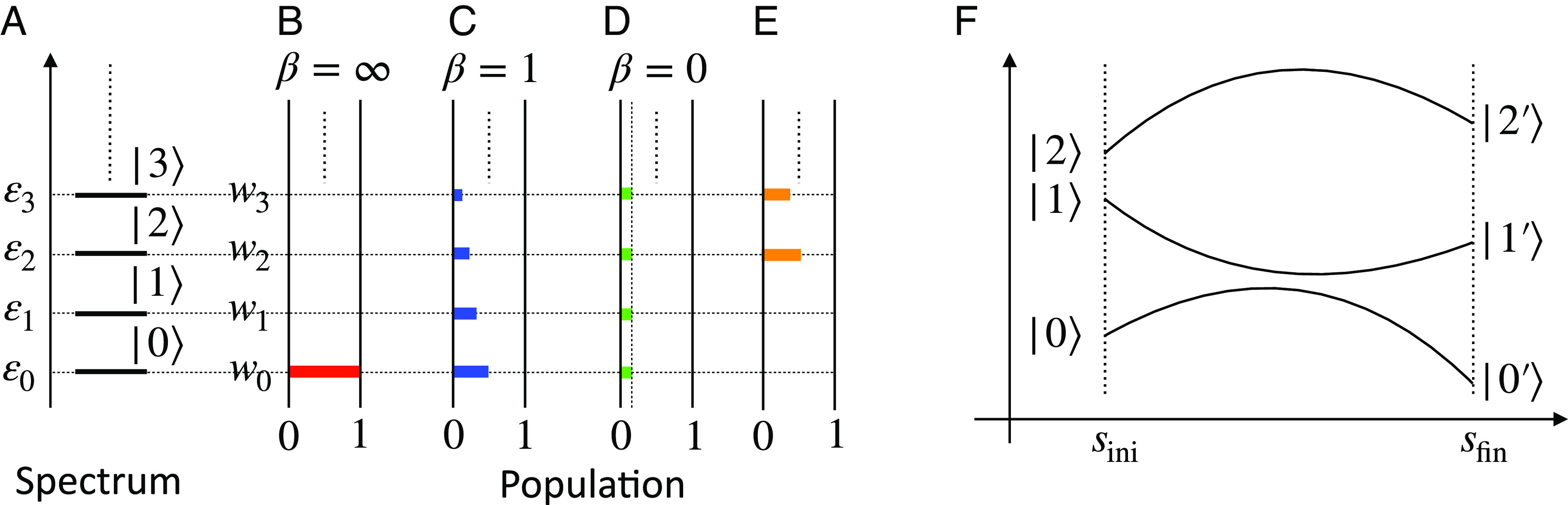
Quantum advantage in VB explained using schematics. (*A*) a typical energy spectrum of $\hat{H}=\sum_{n=0}^{\infty }\varepsilon_{n}\left|n\rangle \langle n\right|$, (*B*, *C*, *D*) populations of the canonical distributions at $\beta =\frac{1}{T}=\infty ,1,0$, respectively (where *T* is the temperature of the bath attached to the system), and (*E*) that of a typical noncanonical distribution. We denote the energy level of |*n*⟩ by *ε*_*n*_ for *n* = 0, 1, 2, … and assume that *ε*_0_ ≤ *ε*_1_ ≤ … ≤ *ε*_*n*_. A mixed state is written as $\hat{\rho }=\sum_{n=0}^{\infty }w_{n}\left|n\rangle \langle n\right|$, where *w*_*n*_ ≥ 0 for *n* = 0, 1, 2, … and $\sum_{n=0}^{\infty }w_{n}=1$. At *β* = ∞, we have *w*_0_ = 1 and *w*_*n*_ = 0 for *n* = 1, 2, … while we have *w*_*n*_ = const. for *n* = 0, 1, 2, … at *β* = 0. (*F*) Schematic of the change of the energy spectrum of $\hat{H}\left(s\right)$ from *s*_ini_ to *s*_fin_. By construction, the optimal solution to the VB problem corresponds to the canonical distribution of the corresponding physical system at *β* = 1. If one could start with the system in a canonical distribution at zero temperature (*β* > > 1), which is the ground state, then one could raise temperature slowly to reach the canonical distribution at *β* = 1 and, hence, obtain the optimum solution to the VB problem. QAVB uses a variant of quantum annealing to approximate the ground state at close to *β* = ∞ and then increases the temperature to *β* = 1, leading to a closer approximation to the canonical distribution. Moreover, it requires only a single run (especially with *s*_0_ = 1 as in Algorithm 1) without any dependence on initialization. In contrast, for other methods based on classical statistics or Monte Carlo methods, the challenge is to start with a canonical distribution at any *β*_0_ < 1 and avoid having to cool the temperature where the system will get stuck in local minima. For example, the deterministic annealing method either starts with a very high initial temperature (where the canonical distribution is trivially known, i.e., uniform) and gets stuck at a saddle point or starts with a random initialization of the distribution at a finite temperature (which would not be the canonical distribution for that temperature) getting easily stuck in local minima and leading to different estimations sensitive to the initial choice.

We next consider the relationship between the populations of the canonical distributions at *β* = ∞ and at *β* = 0 and discuss how such canonical distributions might evolve as *β* is changed adiabatically to *β* = 1. Note that a canonical distribution at *β* = 1 corresponds to an optimal solution to the VB problem. Fig. 1 *B* and *C* show schematic representations of populations of the canonical distributions at *β* = ∞ and at *β* = 1, respectively. As shown in Fig. 1 *B* and *C*, once we get |0; cl⟩, we obtain ${\hat{\rho }}_{*}^{\Sigma ,\theta }\left(\Sigma ,\theta \right)$ in Eq. **10** by pumping up the population of the ground state to those of excited states deterministically. If one starts at a very high temperature (i.e., *β* ≈ 0), as often done in DAVB, then the initial canonical distribution is uniform, and it is well known that when one reduces temperature, then it gets stuck in a saddle point, far from the canonical distribution. On the other hand, if one starts at finite temperature, then one has to assume a noncanonical distribution as the initial condition, and then the algorithm gets easily stuck in a local minimum. In other words, it is difficult to obtain the canonical distribution at *β* = 1 from that at *β* = 0 or a noncanonical distribution, as shown in Fig. 1*E*. Thus, if the ground state is available, it helps us to solve the VB problem, Eq. **1**.

## Quantum Annealing Variational Bayes (QAVB) Inference

We describe QAVB by following ref. 16. In general, QA (18–20) is a method to find the ground state of a given Hamiltonian by using the adiabatic theorem, as shown in Fig. 1*F*. If we can design a parametrized Hamiltonian $\hat{H}\left(s\right)$ such that $\hat{H}\left(s_{\mathrm{ini}}\right)$ is solvable and $\hat{H}\left(s_{\mathrm{fin}}\right)={\hat{H}}_{\mathrm{pr}}^{\theta }+{\hat{H}}_{\mathrm{cl}}^{\Sigma |\theta }$, then one can apply QA to approximate the desired ground state. In the case of QA, the dynamics are described by the Schrödinger equation; then, the adiabatic theorem holds for the dynamics of a time-dependent system. However, in our case, the state evolution follows the MF equation, and a similar property is not known. The analysis of adiabatic evolution in QAVB is one of the goals of this paper and is addressed in a later section.

In the rest of this paper, we formulate QAVB by adding a noncommutative term to the Hamiltonians of VB, Eq. **7** and Eq. **8**, and confirm its validity. By using Eq. **7** and Eq. **8**, we then define the following Gibbs operator:

[13] $$\hat{f}\left(\beta ,s\right):=\exp\left(-{\hat{H}}_{\text{pr}}^{\theta }-\beta \left(1-s\right){\hat{H}}_{\text{cl}}^{\Sigma |\theta }-\beta s{\hat{H}}_{\text{qu}}^{\Sigma }\right).$$

Here, the third term of Eq. **13** is given by ${\hat{H}}_{\mathrm{qu}}^{\Sigma }:=\sum_{i=1}^{N}{\hat{H}}_{\mathrm{qu}}^{\sigma_{i}}$, and each term on the right-hand side satisfies the following noncommutative relation:

[14] $$\left[{\hat{H}}_{qu}^{\sigma_{i}},\left(\overset{i-1}{\underset{j=1}{⊗}}{\hat{I}}^{\sigma_{j}}\right)⊗{\hat{\sigma }}_{i}⊗\left(\overset{N}{\underset{j=i+1}{⊗}}{\hat{I}}^{\sigma_{j}}\right)⊗{\hat{I}}^{\theta }\right]\neq \theta .$$

Here, ${\hat{\sigma }}_{i}$ is a matrix such that ${\hat{\sigma }}_{i}|\sigma_{i}\rangle =\sigma_{i}\left|\sigma_{i}\right\rangle$ and ${\hat{I}}^{\left(\cdot \right)}$ is the identity operator for the corresponding Hilbert space. Using Eq. **11** and Eq. **13**, we consider the following quantum relative entropy:

[15] $$S\left({\hat{\rho }}^{\Sigma ,\theta }\left\|\frac{\hat{f}\left(\beta ,s\right)}{Z\left(\beta ,s\right)}\right.\right):={\text{Tr}}_{\Sigma ,\theta }\left[{\hat{\rho }}^{\Sigma ,\theta }\left\{\ln{\hat{\rho }}^{\Sigma ,\theta }-\ln\frac{\hat{f}\left(\beta ,s\right)}{Z\left(\beta ,s\right)}\right\}\right],$$

where 𝒵(*β*, *s*) is the partition function given by $Z\left(\beta ,s\right):={\mathrm{Tr}}_{\Sigma ,\theta }[\hat{f}\left(\beta ,s\right)]$. By minimizing Eq. **15** with respect to ${\hat{\rho }}^{\Sigma ,\theta }$, we can estimate the distribution of *θ*. However, the minimization problem of Eq. **15** is quite difficult; then, we utilize the following decomposition:

[16] $${\hat{\rho }}^{\Sigma ,\theta }\approx {\hat{\rho }}^{\Sigma }⊗{\hat{\rho }}^{\theta }.$$

Eq. **16** is often called the MF approximation. By performing the variational calculation of Eq. **15** with Eq. **16**, we obtain the following update equations:

[17] $${\hat{\rho }}_{t+1}^{\Sigma }\propto \exp\left({\text{Tr}}_{\theta }\left[\left({\hat{I}}^{\Sigma }⊗{\hat{\rho }}_{t+1}^{\theta }\right)\ln\hat{f}\left(\beta_{t},s_{t}\right)\right]\right),$$

[18] $${\hat{\rho }}_{t+1}^{\theta }\propto \exp\left({\text{Tr}}_{\Sigma }\left[\left({\hat{\rho }}_{t}^{\Sigma }⊗{\hat{I}}^{\theta }\right)\ln\hat{f}\left(\beta_{t},s_{t}\right)\right]\right).$$

Finally, we summarize this algorithm in Algorithm 1. Note that the setting of *s*_0_ = 1 in the algorithm ensures that there is no dependence of the results on the initial choice of ${\hat{\rho }}_{0}^{\Sigma }$, and hence, this variant of the QAVB is executed only once for a given problem. In contrast, for DAVB and VB (also for QAVB where *s*_0_ < 1; ref. 16), results are highly sensitive to the initial conditions and good outcomes are obtained with low probability.


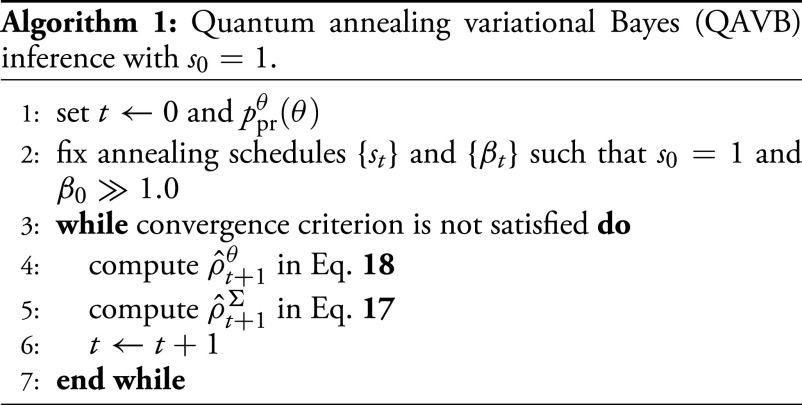


There are multiple candidates for $H_{\text{qu}}^{\Sigma }$ that satisfy Eq. **14**. In numerical simulations, we use the following ${\hat{H}}_{\mathrm{qu}}^{\sigma_{i}}$:

[19] $$\begin{array}{l} {\hat{H}}_{\text{qu}}^{\sigma i}:=\left(\overset{i-1}{\underset{j=1}{⊗}}{\hat{I}}^{\sigma_{j}}\right)⊗\left(\sum_{k=1}^{K}\left(|\sigma_{i}=k\rangle \langle \sigma_{i}=k+1|\right.\right.\\ \;\;\;\;\;\;\;\;\;\;\;\;\;\;\;\;\left.\left.+|\sigma_{i}=k+1\rangle \langle \sigma_{i}=k|\right)\right)⊗\left(\overset{N}{\underset{j=i+1}{⊗}}{\hat{I}}^{\sigma_{j}}\right)⊗{\hat{I}}^{\theta }, \end{array}$$

where |*σ*_*i*_ = *K* + 1⟩=|*σ*_*i*_ = 1⟩. To run QAVB, we also need to fix an annealing schedule; so, it is quite important to construct an efficient one. However, there are an infinite number of possible annealing schedules; so we need to limit ourselves. In ref. 16, the following annealing schedules for *s*_*t*_ and *β*_*t*_ = 1/*T*_*t*_, where *T*_*t*_ is the temperature of the bath to which the system is attached at time *t*, are adopted:

[20] $$S_{t}=S_{0}\times \max\left(1-t/\tau_{1},0.0\right),$$

[21] $$\beta_{t}=\left\{\begin{array}{ll} \beta_{0} & \left(t\leq \tau_{1}\right),\\ 1+\frac{\left(\beta_{0}-1\right)\left(\tau_{2}-t\right)}{\tau_{2}-\tau_{1}} & \left(\tau_{1}\leq t\leq \tau_{2}\right),\\ 1.0 & \left(t\geq \tau_{2}\right). \end{array}\right.$$

**Fig. 2.**
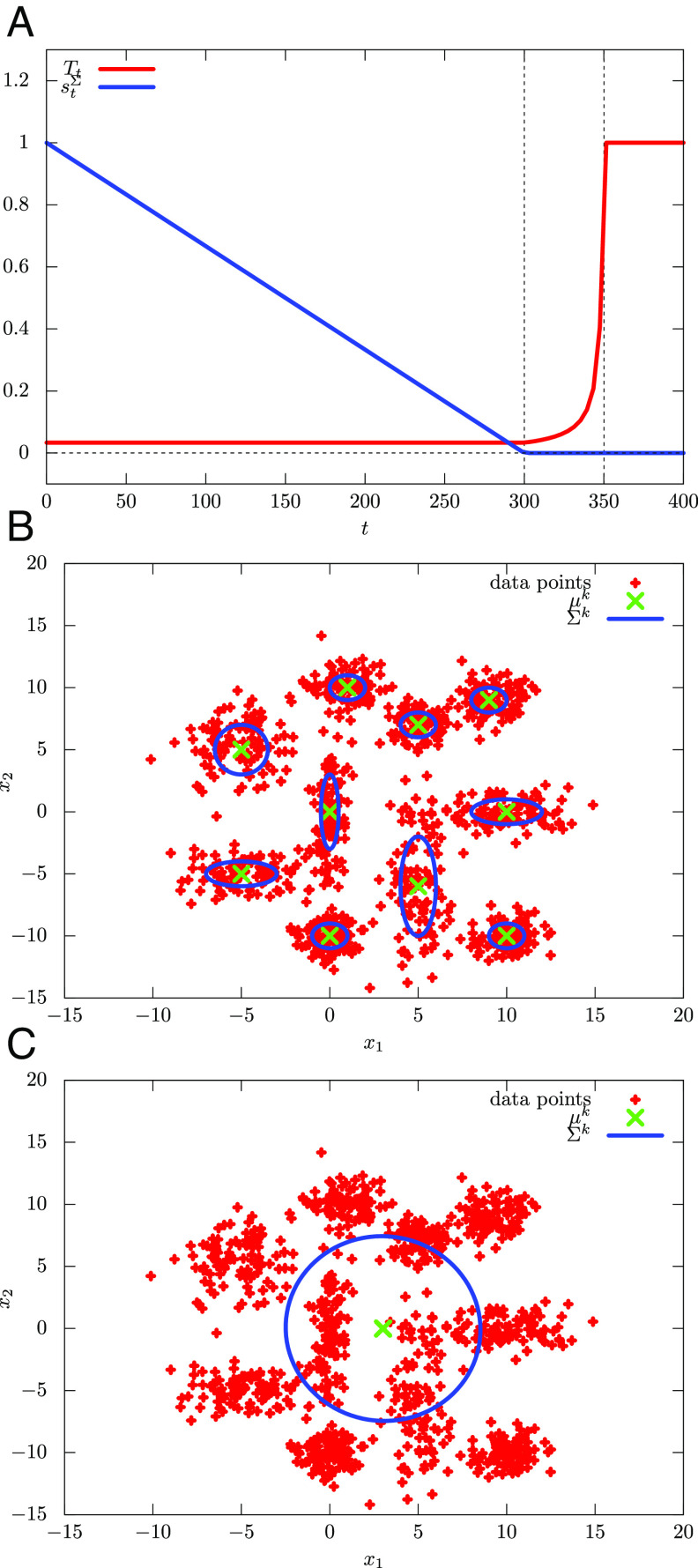
(*A*) Annealing schedules described by Eqs. **20** and **21** with *β*_0_ = 30.0, *s*_0_ = 1.0, *τ*_1_ = 300, and *τ*_2_ = 350. (*B*) Two-dimensional dataset generated by ten Gaussian functions. Each data point has a label. (*C*) Gaussian functions at step 112 estimated by DAVB with *β*_0_ = 0.0010, *s*_0_ = 0.0, *τ*_1_ = 10, and *τ*_2_ = 100. Only one Gaussian mode dominates; the rest have *π*_*i*_ ≈ 0. This shows that when DAVB starts at a high temperature and is slowly cooled, it gets stuck in a saddle point.

Note that Eq. **20** and Eq. **21** are characterized by four parameters: *s*_0_, *β*_0_, *τ*_1_, and *τ*_2_. Furthermore, the performance of QAVB on *s*_0_ and *β*_0_ is investigated in ref. 16, and it shows that *s*_0_ = 1.0 and *β*_0_ = 30.0 are effective. In Fig. 2*A*, we plot the annealing schedules described by Eqs. **20** and **21** with *β*_0_ = 30.0, *s*_0_ = 1.0, *τ*_1_ = 300, and *τ*_2_ = 350.

## Mechanisms of QAVB

To discuss the dynamics of an estimate by QAVB, we focus on the annealing schedule described by Eqs. **20** and **21** with *β*_0_ ≫ 1.0 and *s*_0_ = 1.0 since, as we see later, QAVB with this annealing schedule shows high performance. The annealing schedule can be divided into two parts. First quantum fluctuations are gradually decreased until they disappear at low temperature, and then, the temperature *β* is raised to 1, at which point the cost functions of QAVB and VB are identical. We develop a highly likely mechanism of QAVB on the basis of this decomposition as follows.

Due to the nature of the canonical distribution, the ground state of ${\hat{H}}_{\mathrm{cl}}^{\Sigma |\theta }$ dominates the density operator at finite but large *β* > > 1. In the QA part of the annealing schedule, the state is expected to gradually vary from the ground state of the Hamiltonian, ${\hat{H}}_{\mathrm{qu}}^{\Sigma }$, that has a trivial ground state (by design) to that of the Hamiltonian of interest, ${\hat{H}}_{\mathrm{cl}}^{\Sigma |\theta }$ in Eq. **5**. Of course, given the parameterized form of ${\hat{\rho }}^{\Sigma ,\theta }$ used in VB, and the MF approximation, ${\hat{\rho }}^{\Sigma ,\theta }={\hat{\rho }}^{\Sigma }⊗{\hat{\rho }}^{\theta }$ one can only approximate the ground state. Picking more expressive functional forms or, as shown in the numerical section, increasing the number of clusters *K* in the GMM estimation problem can improve the expressive power and lead to better approximation and improved performance.

Furthermore, the ground state corresponds to the hard clustering assignment, in the sense that each data point is assigned to exactly one categorical value. This follows from the observation that ${\hat{H}}_{\mathrm{cl}}^{\Sigma |\theta }$ is diagonal, and hence, its ground state corresponds to a diagonal element, where Σ is fixed, which implies that each data point is assigned to a single hidden categorical value. Such optimal hard clustering is also an important problem in machine learning, and thus, it is useful to obtain or closely approximate the ground state.

Then, in the second part of the annealing schedule, we raise the temperature to obtain the state that minimizes the cost function of VB, Eq. **15** with *β* = 1.0 and *s* = 0.0. From the viewpoint of physics, saddle points are associated with spontaneous symmetry breaking (SSB). We often come across SSB in the process of decreasing temperature; on the other hand, all the experiments and theoretical analysis so far have shown that there is no SSB in the process of increasing temperature. Thus, we can expect that, if we have the ground state at *T* ≈ 0 (*β* = 1/*T* ≈ ∞), then we can have the canonical distribution at any *β* just by decreasing *β*. This discussion is also expected to hold for QAVB. In this paper, we validate this discussion by looking at the estimates before and after raising the temperature (Table 1).

**Table 1.** Success rates of QAVB at convergence and at the end of the QA part and the best achievable success rates from the generative model used to create datasets

At convergence	At the end of the QA part	Best achievable
0.9221 ± 0.0497	0.8919 ± 0.0496	0.9833 ± 0.0045

We created 10 datasets by using the same generative model used to create Fig. 2 and computed the mean and SD of the performance. We set *K* = 20, *τ*_1_ = 300, and *τ*_2_ = 50. Note that *s*_0_ = 1.0 and *β*_0_ = 30.0. QAVB achieves a high success rate that is close to the success rate of the generative model at convergence. Furthermore, the success rate of QAVB at the end of the QA part is also very close to that at convergence. This demonstrates that QAVB approximates the ground state well and obtains a good hard-clustering solution at the end of the QA step.

DAVB was also developed on the idea that an optimal estimate is continuously connected, and a global optimum would be obtained by changing temperature gradually. The update steps are identical to that of QAVB when *s*_*t*_ = 0; *SI Appendix* for derivations. However, if we start DAVB with high temperature (i.e., *β* ≈ 0), we cannot avoid SSB, and if we start it with low temperature, then the final estimate depends strongly on the initial configuration. Such deficiencies motivated us to develop QAVB and analyze its dynamics. We show here why QAVB has a different dynamics, allowing it to outperform other methods.

## Numerical Simulations

For demonstrating quantum advantage in VB inference and to showcase the dynamics of QAVB, we apply QAVB to the well-known clustering problem using the Gaussian mixture model (GMM). In the numerical simulations, two datasets are investigated: two-dimensional and three-dimensional datasets generated by the GMM^†^. These low-dimensional datasets are sufficient to demonstrate the various factors that contribute to the successful dynamics of QAVB. For applications of QAVB to higher dimensional datasets, please ref. 16. In Fig. 2*B*, the first dataset is shown. To quantify performance, we define the prediction rate, or success rate, as the ratio of how many hidden variables are correctly estimated (i.e., how many data points are assigned to the same Gaussian as in the model that generated the data) to the total number of data points. Note that there is an arbitrariness on the permutation of hidden variables; thus, we use the maximum value with respect to the permutation as the prediction rate.

**Fig. 3.**
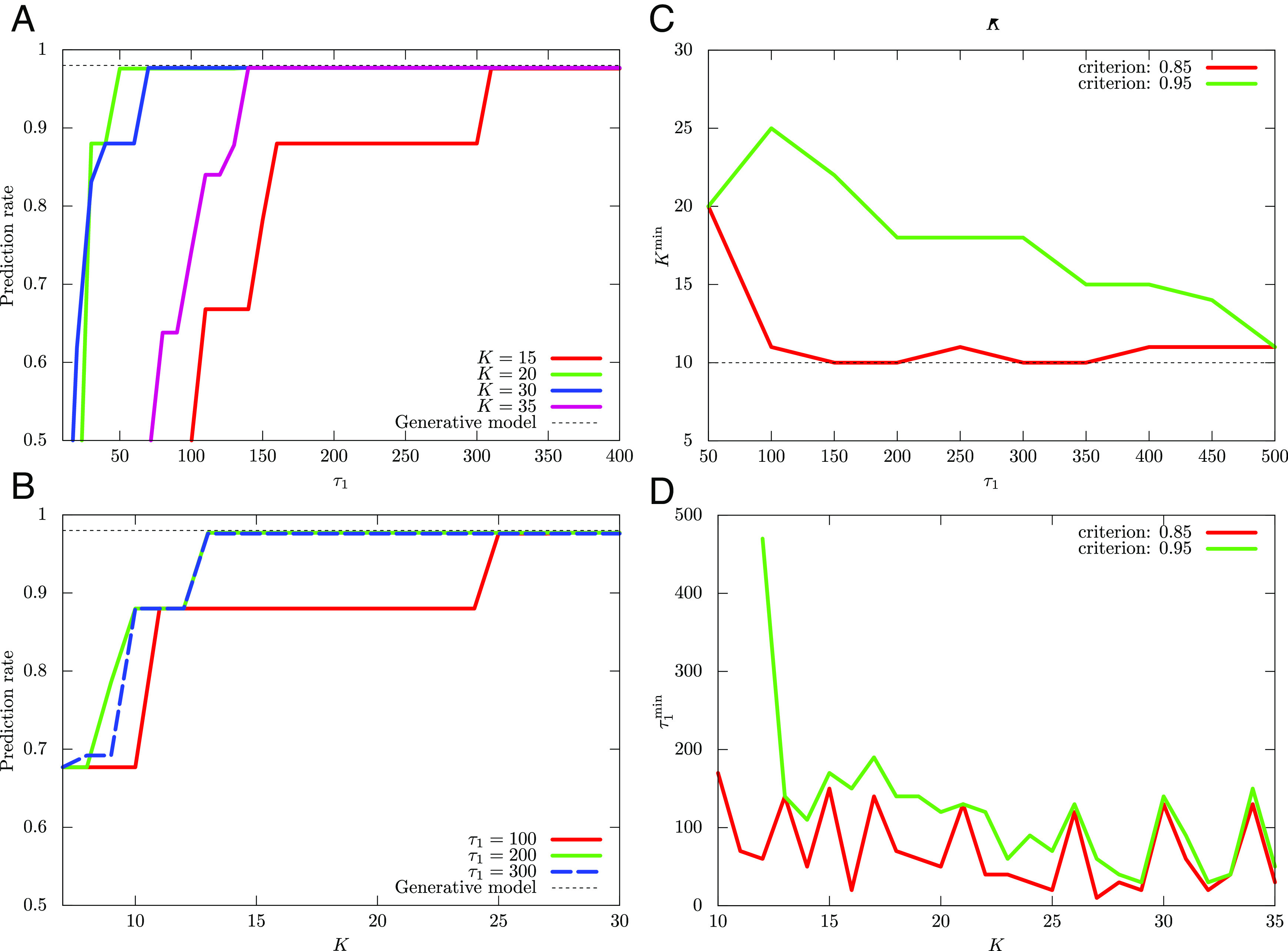
Tradeoffs between quantum annealing duration *τ*_1_ and *K*, the number of Gaussians in the QAVB algorithm: In order for QAVB to achieve a state close to the ground state at zero temperature, both *K*—which determines the expressive power of the MF variational function—and *τ*_1_—which determines how slow we anneal—need to be set. As defined on page 6, for a fixed *τ*_1_, $p_{\text{suc}}^{K}\left(K,\tau_{1}\right)=\max_{K\leq K}p_{\text{suc}}\left(k,\tau_{1}\right)$, that is, the maximum accuracy obtained by varying the number of clusters up to *K* for a fixed *τ*_1_. Similarly, $p_{\text{suc}}^{\tau_{1}}\left(K,\tau_{1}\right)$ is the maximum accuracy obtained for any ${\tau^{\prime }}_{1}\leq \tau_{1}$ for any fixed *K*. Numerical computations for the two-dimensional data illustrated in Fig. 2 are presented here; for this dataset, the maximum possible success rate as obtained from the generative model is 0.98 and is shown by the black dotted lines. (*A*) Dependence of $p_{\text{suc}}^{K}\left(K,\tau_{1}\right)$ on *τ*_1_ for different *K*’s, (*B*) that of $p_{\text{suc}}^{\tau_{1}}\left(K,\tau_{1}\right)$ on *K*. As these plots show, QAVB can achieve almost optimal performance for a wide range of *K* ≥ 14 and 100 ≤ *τ*_1_ ≤ 300; two best-performing combinations are (*K* = 20, *τ*_1_ = 50) and (*K* = 14, *τ*_1_ = 200). Next, we set a target success rate, *p*_cr_, of 0.85 and 0.95, respectively. (*C*) *K*^min^(*τ*_1_), which is the minimum value of *K* to achieve a given *p*_cr_, as *τ*_1_ is varied, and (*D*) $\tau_{1}^{\min}\left(K\right)$, which is the minimum value of *τ*_1_ to achieve a given *p*_cr_. For example, for *τ*_1_ = 500, one can match the best performance for *K* = 12. Note that in (*C*) and (*D*), the values are not monotonically decreasing. Note that when *K* is set larger than 10 (actual number of Gaussians), the posterior probabilities of only 10 modes match the corresponding ground-truth values, and the rest of the *K* − 10 modes have almost zero posterior probabilities. Similar results are shown for a 3-D dataset in Fig. 6.

We use Eqs. **20** and **21** and set *β*_0_ = 30.0 and *s*_0_ = 1.0 for the annealing schedule of the experiments. With these settings, Algorithm 1is run only once for any given choices of *K*, *τ*_1_, and *τ*_2_. The prediction rate is a function of the hyperparameters *K* and *τ*_1_, and we hereafter denote it by *p*_suc_(*K*, *τ*_1_). Clearly, the duration of the QA steps, *τ*_1_, determines how closely one can track the ground state and plays a crucial role. The number of clusters, *K*, determines the number of parameters and, hence, the expressive power of ${\hat{\rho }}^{\Sigma ,\theta }$ in approximating the ground state. one of the goals of our experiments is to show the tradeoffs between these two hyperparameters. Since *p*_suc_(*K*, *τ*_1_) is not monotonic with respect to *K* and *τ*_1_, we define $p_{\text{suc}}^{K}\left(K,\tau_{1}\right):=\max_{K^{\prime }\leq K}p_{\text{suc}}\left(K^{\prime },\tau_{1}\right)$ and $p_{\text{suc}}^{\tau_{1}}\left(K,\tau_{1}\right):=\max_{{\tau^{\prime }}_{1}\leq \tau_{1}}p_{\text{suc}}\left(K,{\tau^{\prime }}_{1}\right)$. In Fig. 3 *A* and *B*, we plot the dependence of $p_{\text{suc}}^{K}\left(K,\tau_{1}\right)$ on *τ*_1_ and the dependence of $p_{\text{suc}}^{\tau_{1}}\left(K,{\tau^{\prime }}_{1}\right)$ on *K*, respectively. These results show that for sufficiently large *τ*_1_ and *K*, QAVB shows a high prediction rate that almost matches the upper bound set by the generative model, with full knowledge. Given prediction criterion *p*_cr_, we then define $K^{\min}(\tau_{1}):=\arg\min_{K}p_{\text{suc}}\left(K,\tau_{1}\right)$ and $\tau_{1}^{\min}\left(K\right):=\arg\min_{\tau_{1}}p_{\text{suc}}\left(K,{\tau^{\prime }}_{1}\right)$ subject to *p*_suc_(*K*, *τ*_1_)≥*p*_cr_. In Fig. 3 *C* and *D*, we plot *K*^min^(*τ*_1_) and *τ*_1_^min^(*K*), respectively. These figures show that, to achieve *p*_cr_ = 0.85, 0.95, relatively small *K* and *τ*_1_ are enough.

**Fig. 4.**
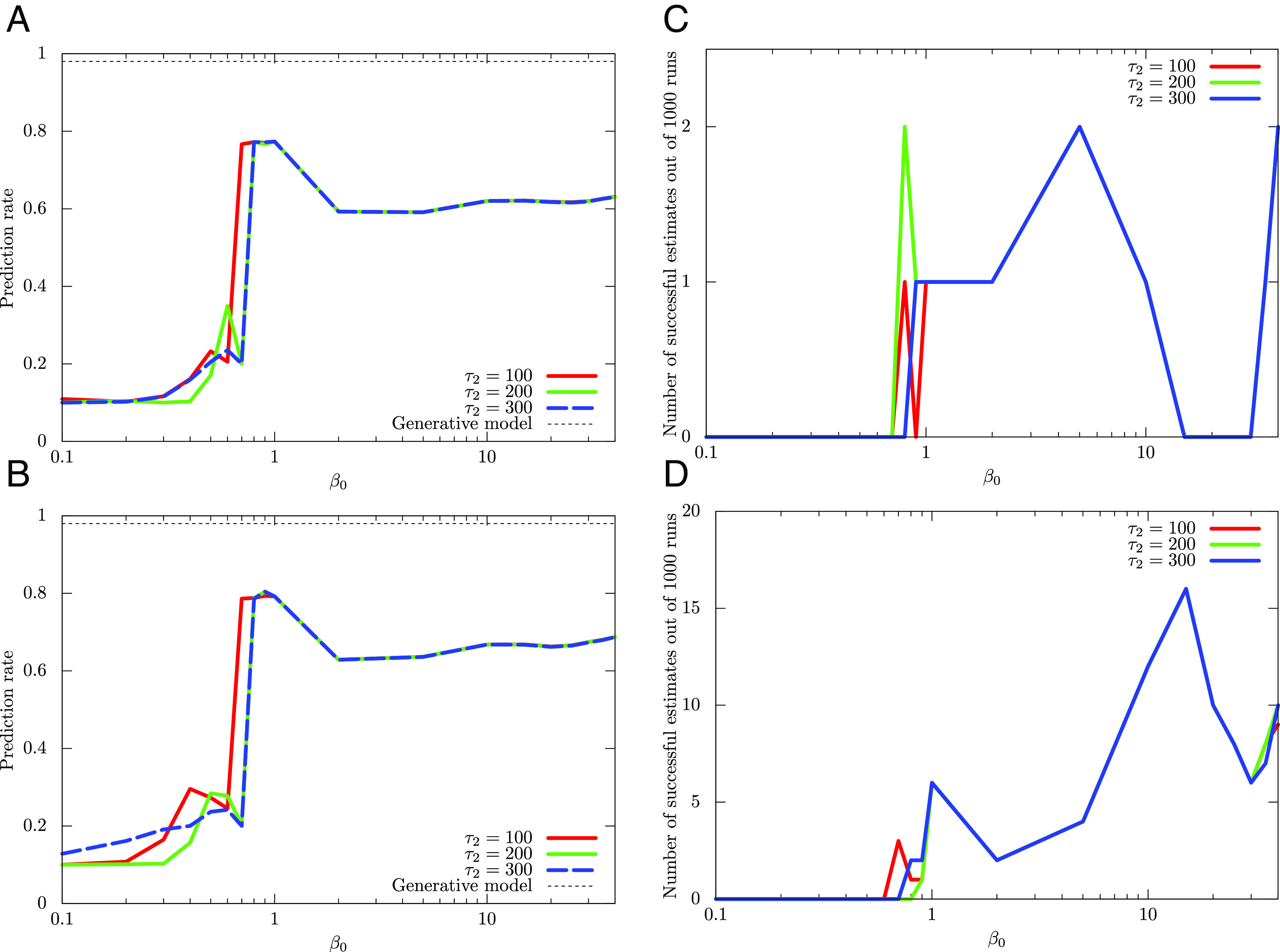
Comparative average performance of DAVB and its dependence on the initial temperature *β*_0_. Note that in DAVB, the performance depends strongly on the initial distribution, i.e., ${\hat{\rho }}_{0}^{\Sigma }$. For the two-dimensional dataset, we profile the dependence of the average prediction rate of DAVB on *β*_0_ for (*A*) *K* = 20 and (*B*) *K* = 30. The number of times that achieves *p*_cr_ = 0.95 out of 1,000 runs for (*C*) *K* = 20 and (*D*) *K* = 30 is also plotted. As is expected, the average performance is inferior to that of QAVB, which for a wide range of choices of parameters gives a near-optimal result in a single run.

Next, we turn our attention to DAVB. We again use Eq. **21** for *β*_*t*_ in DAVB and set *τ*_1_ = 10 and *τ*_2_ = 100, 200, 300. In Fig. 4 *A* and *B*, we plot the dependence of the average prediction rate of DAVB on *β*_0_ for *K* = 20, 30, respectively. And, in Fig. 4 *C* and *D*, we also plot the number of times that achieves *p*_cr_ = 0.95 on *β*_0_ for *K* = 20, 30, respectively. These figures show that the average prediction rate of DAVB is much lower than that of QAVB, and DAVB rarely achieves *p*_cr_ = 0.95.

**Fig. 5.**
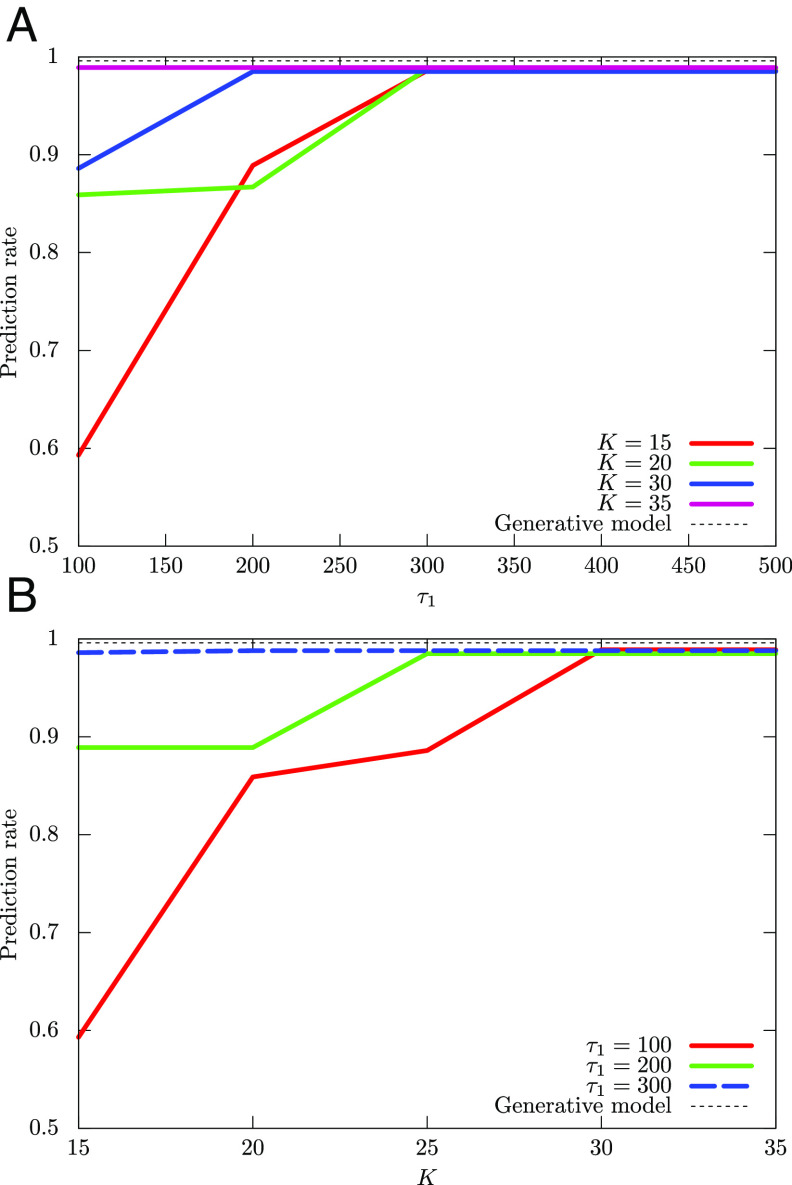
Results paralleling that of Fig. 3 are shown here for a 3-D dataset: (*A*) Dependence of $p_{\text{suc}}^{K}\left(K,\tau_{1}\right)$ on *τ*_1_ and (*B*) that of $p_{\text{suc}}^{\tau_{1}}\left(K,\tau_{1}\right)$ on *K*. These exhibit very similar tradeoffs observed in Fig. 3 and get very close to the optimal performance obtained from the ground-truth generative model used to create the dataset (shown by the black dotted line).

We next present numerical results on a three-dimensional dataset. In Fig. 5, we plot the dependence of $p_{\text{suc}}^{K}\left(K,\tau_{1}\right)$ on *τ*_1_ and the dependence of $p_{\text{suc}}^{\tau_{1}}\left(K,\tau_{1}\right)$ on *K*, respectively.

**Fig. 6.**
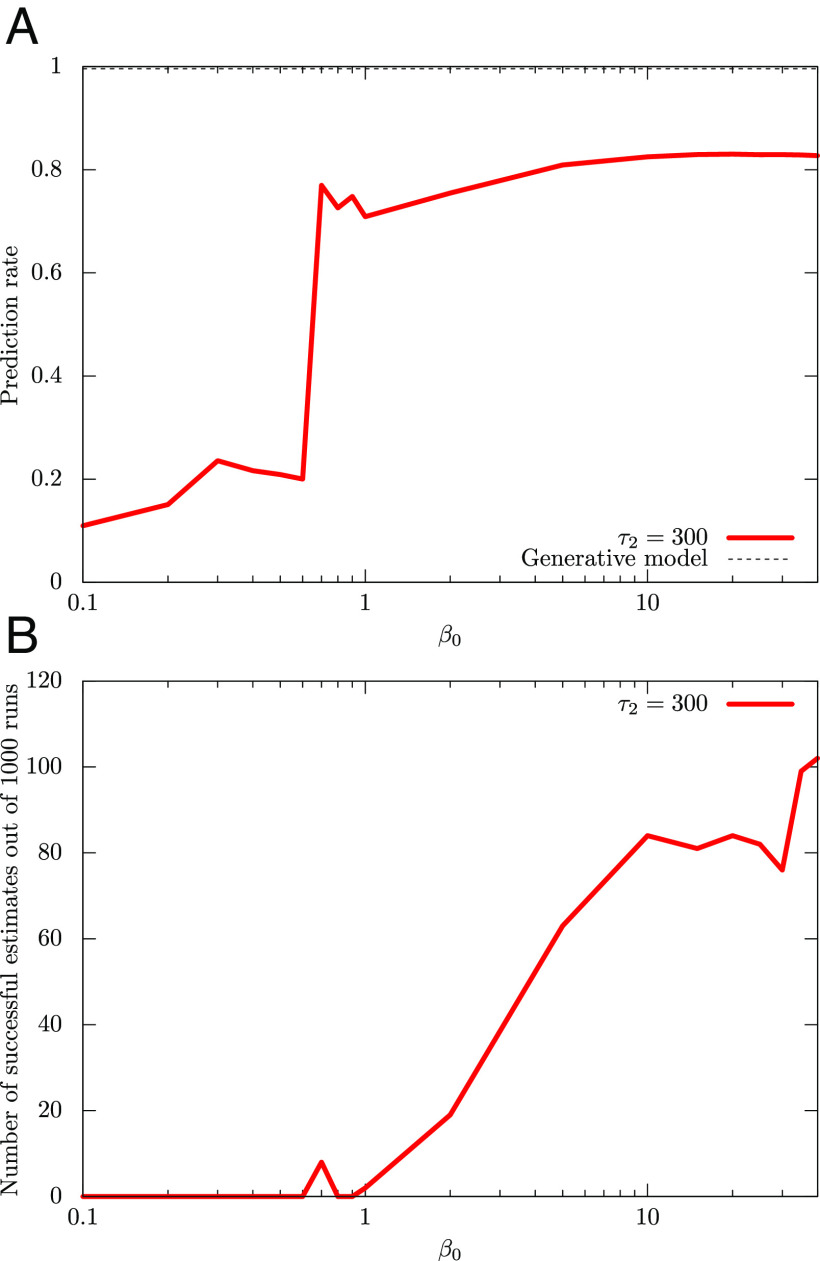
Performance metrics of DAVB for the 3-D data analyzed in Fig. 5 are shown here: (*A*) Dependence of the average prediction rate of DAVB on *β*_0_. (*B*) Number of times that achieves a prediction rate of 0.95 out of 1,000 runs. We set *K* = 20. A single run of QAVB can outperform DAVB. To get a relatively good performance from DAVB, one needs to start at a low temperature *β*_0_ > > 1 and increase it to *β* = 1; even then, the probability of getting a good prediction rate is low.

In Fig. 6 *A* and *B*, we plot the dependence of the average prediction rate of DAVB and the number of times that achieves *p*_cr_ = 0.95 on *β*_0_, respectively. Here, we set *τ*_1_ = 10, *τ*_2_ = 300, and *K* = 20.

The numerical result on the three-dimensional dataset is consistent with the case of the two-dimensional dataset though it is quantitatively different from the case of the two-dimensional dataset.

**Fig. 7.**
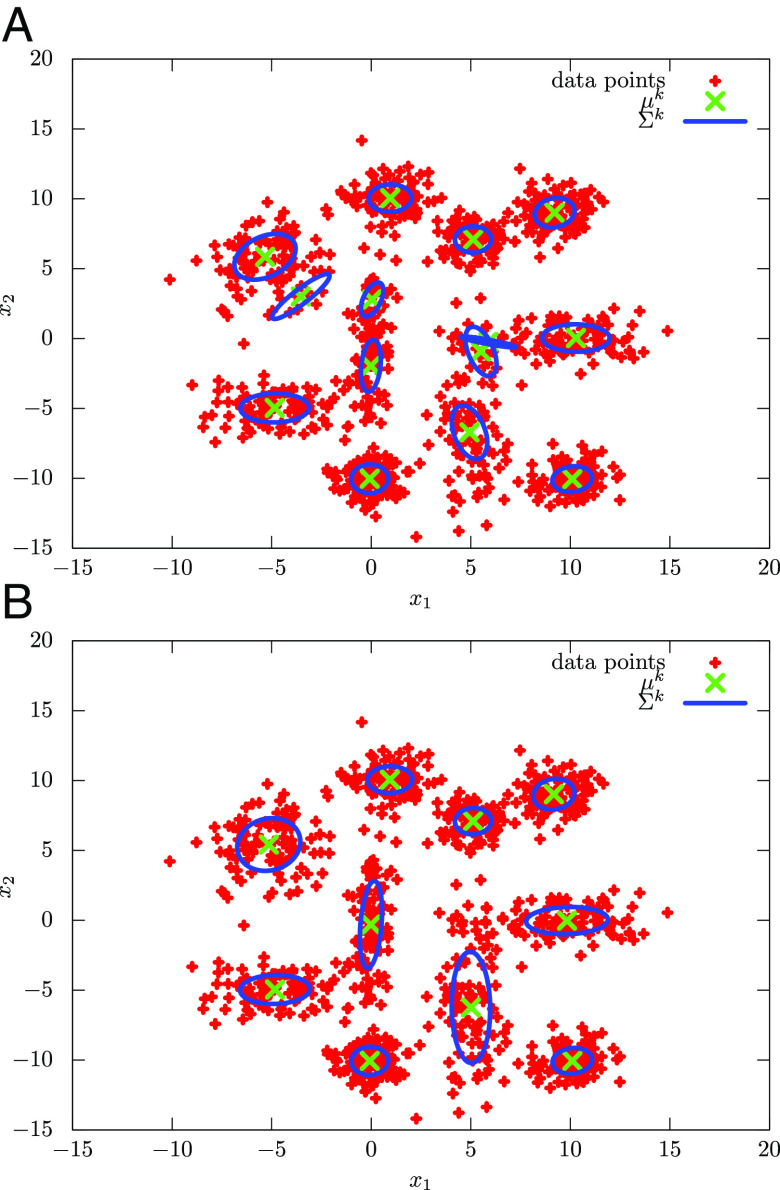
GMM estimates at the end of the QA step in QAVB and the critical role played by the QA part: For the 2-D dataset in Fig. 2*B*, we visualize the estimated Gaussians functions, when *β*_0_ = 30, and *K* = 20 (*A*) at step 300 (*β*_300_ = 30.0), i.e., at the end of the QA step, and (*B*) at step 460 (*β*_460_ = 1.0), i.e., at the end of the QAVB algorithm. Only the Gaussian functions whose weight is greater than 0.01 are shown; i.e., when the probability of picking a Gaussian, *π*_*j*_ > 0.01, then the *j*-th Gaussian function is shown for *j* = 1, 2, …, *K*. This shows that by the end of the QA step, the algorithm has found an almost-optimal solution, and increasing temperature only fine-tunes these estimates. This is further borne out by the results presented in Table 1.

To understand the dynamics of QAVB, it is instructive to study cluster assignments of QAVB at the end of the QA part of the annealing schedule at *t* = *τ*_1_ and at convergence. We show the cluster assignments of QAVB with *β*_0_ = 30.0 in Fig. 7 *A* and *B*. The estimates at the end of the QA part are almost same as those at convergence. Only a few of the clusters in the ground-truth data are split. Thus, the process of raising temperature is not that important. This is expected due to the absence of SSB, and it is quite reasonable to focus on the QA part. Fig. 7 *A* and *B* show that, in the case of low temperature, QAVB successfully estimates the ground state, while QAVB does not in the case of high temperature. These results are also consistent with the discussions of the possible mechanism of QAVB.

In Table 1, we show the success rates of QAVB at convergence and at the end of the QA part and the best possible success rates with full knowledge of the generative model. Ten datasets were created by using the same generative model to create the dataset shown in Fig. 2, and then the mean and SD of the performance were computed. The success rate of QAVB at convergence is very close to that of the generative model, and that of QAVB at the end of the QA part is also very close to them. This observation reflects two points. The first one is simply that QAVB is successful for the generative model under consideration. The second one is that the soft clustering defined via the minimization problem of the KL divergence, which is Eq. **10** at *β* = 1 and *s* = 0, and the hard clustering defined via that at *β* ≫ 1 are similar. In the case of the hard clustering problem, however, the data points are classified into a larger number of clusters. That is, out of the *K* Gaussians (*K* > 10 where the generative model has 10 Gaussians), more than 10 have *π*_*j*_ > 0.01. Thus, the success rate of QAVB at the end of the QA part is slightly worse than at convergence.

## Adiabatic-Theorem-like Property: Similarity and Differences Between QA and QAVB

In QA, the total Hamiltonian is constructed by the convex combination of a Hamiltonian that describes an optimization problem of interest and a noncommutative Hamiltonian that can be easily diagonalized. Similar to QA, we construct Eq. **13** by the convex combination of two Hamiltonians. On the other hand, the main difference is that QA solves the Schrödinger equation, but QAVB solves the MF equation. Thus, the adiabatic theorem (17) does not directly hold. We next analytically examine QAVB and discuss an adiabatic-theorem-like property of QAVB. First, let us consider the eigenvalue decomposition of Eq. **13**:

[22] $$\ln\hat{f}\left(\beta ,s\right)=\sum_{n=0,1,2,\ldots }\varepsilon_{n}\left(\beta ,s\right)|n;\beta ,\;s;\;\Sigma ,\theta \rangle \langle n;\beta ,s;\Sigma ,\theta |.$$

Here, *ε*_*n*_(*β*, *s*) is the (*n* + 1)-th largest eigenvalues with *β* and *s* for *n* = 0, 1, 2, …. As explained before, QAVB is based on the MF theory; then, it is quite natural to consider the MF approximated form of Eq. **22**:

[23] $$\begin{array}{l} \ln{\hat{f}}^{\text{MF}}\left(\beta ,s\right)=\sum_{n=0,1,2,\ldots }\varepsilon_{n}^{\text{MF}}\left(\beta ,s\right)|n;\beta ,s;\Sigma \rangle \langle n,\beta ,s;\Sigma |\\ \;\;\;\;\;\;\;\;\;\;\;\;\;\;\;\;\;\;\;\;\;\;\;\;\;\;\;\;\;\;\;\;\;⊗|n;\beta ,s;\theta \rangle \langle n;\beta ,s;\theta |. \end{array}$$

Here, *ε*_*n*_^MF^(*β*, *s*) is the (*n* + 1)-th largest MF eigenvalues with *β* and *s* for *n* = 0, 1, 2, …, and |*n*; *β*, *s*; Σ⟩⊗|*n*; *β*, *s*; *θ*⟩ is the eigenvectors associated with *ε*_*n*_^MF^(*β*, *s*). By using Eq. **23**, the update equations of QAVB, Eqs. **17** and **18**, are rewritten as

[24] $$\ln{\hat{\rho }}_{t+1}^{\Sigma }={\text{Tr}}_{\theta }\left[\left({\hat{I}}^{\Sigma }⊗{\hat{\rho }}_{t+1}^{\theta }\right)\ln{\hat{f}}^{\text{MF}}\left(\beta_{t},s_{t}\right)\right]+\;\text{const}\text{.,}$$

[25] $$\ln{\hat{\rho }}_{t+1}^{\theta }={\text{Tr}}_{\Sigma }\left[\left({\hat{\rho }}_{t}^{\Sigma }⊗{\hat{I}}^{\theta }\right)\ln{\hat{f}}^{\text{MF}}\left(\beta_{t},s_{t}\right)\right]+\;\text{const}\text{.,}$$

Assuming that ${\hat{\rho }}_{t}^{\Sigma }=|0;\beta_{t-1},s_{t-1};\Sigma \rangle \langle 0;\beta_{t-1},s_{t-1};\Sigma |$ and ⟨0; *β*_*t* − 1_, *s*_*t* − 1_; Σ|0; *β*_*t*_, *s*_*t*_; Σ⟩≈1, Eq. **25** becomes

[26] $$\begin{array}{l} \ln{\hat{\rho }}_{t+1}^{\theta }={\text{Tr}}_{\Sigma }\left[\left(|0;\beta_{t-1},s_{t-1};\Sigma \rangle \langle 0;\beta_{t-1},s_{t-1};\Sigma |⊗{\hat{I}}^{\theta }\right)\right.\\ \;\;\;\;\;\;\;\;\;\;\;\;\;\;\;\;\;\;\;\;\times \sum_{n}\varepsilon_{n}^{\text{MF}}\left(\beta ,s\right)|n;\beta_{t},s_{t};\Sigma \rangle \langle n;\beta_{t},s_{t};\Sigma |\\ \;\;\;\;\;\;\;\;\;\;\;\;\;\;\;\;\;\;\;\left.⊗|n;\beta_{t},s_{t};\theta \rangle \langle n;\beta_{t},s_{t};\theta |\right]+\;\text{const}\text{.} \end{array}$$

[27] $$\begin{array}{l} \;\;\;\;\;\;\;\;\;\;\;\;\;\;\;\;\;\;\;\;\approx {\text{Tr}}_{\Sigma }\left[\left(|0;\beta_{t-1},s_{t-1};\Sigma \rangle \langle 0;\beta_{t-1},s_{t-1};\Sigma |⊗{\hat{I}}^{\theta }\right)\right.\\ \;\;\;\;\;\;\;\;\;\;\;\;\;\;\;\;\;\;\;\;\;\;\;\;\;\;\times \varepsilon_{0}^{\text{MF}}\left(\beta ,s\right)|0;\beta_{t},s_{t};\Sigma \rangle \langle 0;\beta_{t},s_{t};\Sigma |\\ \;\;\;\;\;\;\;\;\;\;\;\;\;\;\;\;\;\;\;\;\;\;\;\;\;\;\left.⊗|0;\beta_{t},s_{t};\theta \rangle \langle 0;\beta_{t},s_{t};\theta |\right]+\text{const}\text{.} \end{array}$$

[28] $$\;\;\;\;\;\;\;\;\;\;\;\;\;\;\;\;\;\;\;\;\text{=}|0;\beta_{t},s_{t};\theta \rangle \langle 0;\beta_{t},s_{t};\theta |+\text{const}\text{.,}$$

and a similar computation can also be done for Eq. **24**. Note that a constant multiple does not affect the physical property of ${\hat{\rho }}_{t+1}^{\theta }$. In the numerical simulations of QAVB, we varied *s* at fixed *β* at the first QA part; then, the above assumption is reasonable. Thus, the discussion here analytically gives the reason why QAVB shows high performance. In particular, it explains the mechanism by which QAVB gives the ground state of ${\hat{H}}_{\mathrm{cl}}^{\Sigma |\theta }$ in Eq. **5**.

## Time Complexity of QAVB and QAVB as Quantum Dynamics

The main focus of this paper is to quantify how much better QAVB can perform compared with VB and provide analytical results on its dynamics. From the viewpoint of practical applications, its computational complexity is also an important metric. In the case of a classical computer, the time complexity of VB with respect to the number of clusters *K* is *O*(*K*) since VB has single loops on *K*. On the other hand, QAVB requires one to compute the exponentials of *K* × *K* matrices; thus, the time complexity of QAVB with respect to *K* is *O*(*K*^3^). Note that, similarly to VB, the time complexity of QAVB with respect to the number of data points is *O*(*N*); thus, it practically works on a classical computer.

The above situation changes if we assume a quantum computer. The simulations of a quantum system does not increase time complexity compared with that of a classical system. Furthermore, if we can find a local Hamiltonian that describes QAVB, then we can expect a quantum speedup with respect to *K* (21). From this viewpoint, it is worth considering physical implementations of QAVB. From Eqs. **17** and **18**, the relationship between ${\hat{\rho }}_{t}^{\Sigma }$ and ${\hat{\rho }}_{t+1}^{\Sigma }$ is written as

[29] $$\begin{array}{l} {\hat{\rho }}_{t+1}^{\Sigma }=\frac{1}{Z_{t+1}}\exp\left({\text{Tr}}_{\theta }\left[\left({\hat{I}}^{\Sigma }\right.\right.\right.\\ \;\;\;\;\;\;\;\;\;\;\;\;\;\;\left.\left.\left.⊗\exp\left({\text{Tr}}_{\Sigma }\left[\left({\hat{\rho }}_{t}^{\Sigma }⊗{\hat{I}}^{\theta }\right)\ln\hat{f}\left(\beta_{t},s_{t}\right)\right]\right)\right)\ln\hat{f}\left(\beta_{t},s_{t}\right)\right]\right), \end{array}$$

where

[30] $$\begin{array}{l} Z_{t+1}={\text{Tr}}_{\Sigma }\left[\exp\left({\text{Tr}}_{\theta }\left[\left({\hat{I}}^{\Sigma }\right.\right.\right.\right.\\ \left.\left.\left.\left.\;\;\;\;\;\;\;\;\;\;\;\;\;\;\;⊗\exp\left({\text{Tr}}_{\Sigma }\left[\left({\hat{\rho }}_{t}^{\Sigma }⊗{\hat{I}}^{\theta }\right)\ln\hat{f}\left(\beta_{t},s_{t}\right)\right]\right)\right)\ln\hat{f}\left(\beta_{t},s_{t}\right)\right]\right)\right]. \end{array}$$

In Eq. **29**, two types of operations are involved: the exponential operation and partial trace. We show that these two operators are both CPTP because it can be realized in a physical process (22).

First, we discuss that the exponential map is CP when the input density operator is positive semidefinite. Here, we basically follow ref. 23. It is enough to say that the composite operator exp ° *A*_*n*_ is positive for *n* = 1, 2, …, where *A*_*n*_ is an arbitrary *n*-dimensional operator. As described in ref. 23, the family of positive definite operators is closed under point-wise addition and point-wise multiplication; thus, exp ° *A*_*n*_ is positive for *n* = 1, 2, …; thus, the exponential map is completely positive. Note that a Hamiltonian is not necessarily positive semidefinite, but we can always add a constant shift such that the Hamiltonian becomes positive semidefinite. The map of interest is TP because of the partition function, though the exponential map itself is not TP.

Next, we turn our attention to partial trace. We can say that partial trace operation is completely positive by constructing a Kraus operator directly. Let us consider ${\hat{K}}_{\alpha }:={\hat{I}}_{A}⊗\left\langle \alpha \right|$. In general, a density operator for subsystems A and B has the form ${\hat{\rho }}_{AB}:=\sum_{ij\mu \nu }\lambda_{ij\mu \nu }\left|i\rangle \langle j\right|⊗\left|\mu \rangle \langle \nu \right|$, and taking partial trace with respect to subsystem B yields ${\mathrm{Tr}}_{B}\left[{\hat{\rho }}_{AB}\right]=\sum_{\alpha }\sum_{ij\mu \nu }\lambda_{ij\mu \nu }\left|i\rangle \langle j\right|\left\langle \alpha |\mu \right\rangle \left\langle \nu |\alpha \right\rangle =\sum_{ij\mu }\lambda_{ij\mu \mu }\left|i\rangle \langle j\right|$. On the other hand, ${\hat{K}}_{\alpha }$ leads to $\sum_{\alpha }{\hat{K}}_{\alpha }{\hat{\rho }}_{AB}{\hat{K}}_{\alpha }^{†}=\sum_{ij\mu }\lambda_{ij\mu \mu }\left|i\rangle \langle j\right|={\mathrm{Tr}}_{B}\left[{\hat{\rho }}_{AB}\right]$. Thus, we have shown that ${\hat{K}}_{\alpha }$ is the Kraus operator for partial trace. For more details, refer to ref. 22. Thus, we have shown that Eq. **29** is CPTP. In other words, QAVB is physically implementable.

## Discussions

In this paper, we have analyzed the dynamics of QAVB by developing an analytical framework and providing numerical simulations to support the analytical results. In particular, we developed a theoretical framework to understand why there is a quantum advantage in variational bayesian inference. Next, via numerical analysis, we confirmed that the QA part of QAVB is essential by showing that an estimate at the end of the QA part is almost the same as an estimate at convergence at finite temperature. Thus, an optimal solution to the VB problem is essentially obtained at the end of the QA part, and increasing temperature does not affect the estimates very much. We also showed that the estimate at the end of the QA part of the annealing part corresponds to the hard clustering assignment. Second, we developed an adiabatic-theorem-like result that shows that the QA also holds in the case of the MF dynamics. Then, we explained that this generalized QA framework is why QAVB is efficient and gives a quantum advantage. Finally, we discussed the physical realizability of QAVB by showing that QAVB can be expressed as a CPTP map. This discussion tells us that QAVB can be realized in a quantum system. We expect this work to motivate physics-inspired algorithms and further research on emerging fields at the intersection of physics and machine learning.

We have provided physics-based arguments for the quantum advantage in variational Bayes inference, and we have left a rigorous mathematical proof as future work. Rigorous mathematical proofs for recently proposed algorithms which are widely considered to have quantum advantage are also lacking. For example, the quantum approximate optimization algorithm (QAOA) (24) and the variational quantum eigensolver (VQE) (25), which are considered to be equivalent to each other, have attracted much attention as methods to efficiently utilize NISQ devices, and a large number of their variants have been proposed. The main theoretical support for them is that, for *N* → ∞, the QAOA realizes the adiabatic evolution, where *N* is the number of layers in a circuit (24). However, the proof cannot be applied to the QAOA of finite layers; in other words, their computational advantage is not known for the practical setup. As discussed in this paper, the proposed algorithm is expected to have a practical advantage for ML.

## Supplementary Material

Appendix 01 (PDF)

## Data Availability

All study data are included in the article and/or *SI Appendix*. The code for the algorithms proposed in this paper and the code used for generating data can be found at the following github link: https://github.com/hmiyahara512/QAVB (26).

## Footnotes

^*^ *SI Appendix, section 2 for the details of the canonical distribution.*

^†^*SI Appendix*, section 3) document for the details of GMM. The prior and posterior distributions for the GMM are described in *SI Appendix*, section 4.
