# Supplementary material for: Quantum advantage in variational Bayes inference
Source: Proc Natl Acad Sci U S A. 2023 Jul 25;120(31):e2212660120. doi: 10.1073/pnas.2212660120 (PMC10400996; doi:10.1073/pnas.2212660120)
Supplement: Supplementary file 1 — Appendix 01 (PDF) [file pnas.2212660120.sapp.pdf]

Supplemental Information
# – Quantum Advantage in Variational Bayes Inference –

(Dated: February 28, 2023)

This document comprises supplemental information for the paper titled, "Quantum Advantage in Variational Bayes Inference ."

CONTENTS

S-I. Introduction 1

S-II. Canonical distribution 1

S-III. Gaussian mixture model 2

S-IV. Prior and posterior distributions of the GMM 3
A. Prior distributions of the GMM 3
1. Prior distribution of π 3
2. Prior distributions of μ and Λ 3
B. Posterior distribution of Σ in VB 4
C. Posterior distributions of θ in VB 5
1. Posterior distribution of π 5
2. Posterior distributions of μ and Λ 6

S-V. Posterior distribution in DAVB 6
A. Quick review of DAVB 6
B. Posterior distribution of σ_i in DAVB 7
C. Posterior distribution of θ in DAVB 7
1. Posterior distribution of π in DAVB 7
2. Posterior distributions of μ and Σ in DAVB 7

S-VI. Posterior distribution in QAVB 8
A. Posterior distribution of σ_i in QAVB 9
B. Posterior distribution of θ in QAVB 9
C. Definitions of Quantum states and operators of θ 9
1. Posterior distribution of π in QAVB 10
2. Posterior distributions of μ and Σ in QAVB 10

S-VII. Quantization of θ in QAVB 11
A. Generalized version of QAVB 11
B. Why we do not consider the generalized version of QAVB 12

S-VIII. QAVB in the path integral formulation 12
A. The quantum relative entropy and update equations of QAVB in the path integral formulation 12
B. A prior attempt at designing a QA extension of VB using path integral formulation 14

S-IX. Application to real data 15

References 15

S-I. INTRODUCTION

This supplemental information (SI) covers background material such as, the Gaussian Mixture Model (GMM), and the conjugate priors used for various parameters in the GMM. It also covers the update equations that result from using the mean field approximation in varitional Bayes (VB). In addition, it introduces concepts from quantum mechanics, statistical mechanics, and quantum statistical mechanics that would make them more accessible to researchers with

backgrounds in Machine Learning and AI. It also introduces the framework of canonical distributions, and how it leads to deterministic annealing (DA) and DAVB. Finally, it introduces a path integral formulation of QAVB. Some of these background concepts are covered in Refs. [1, 2] , and we have provided a more integrated view in this SI.

S-II. CANONICAL DISTRIBUTION

Let us consider N_{tot} systems together and assume that the total energy of the systems is E_{tot}. We denote, by N_k, the number of the systems in the k-th energy level for $n = 0, 1, 2, \ldots, \infty$; then we have the following constraints:

$$N_{\mathrm{tot}} = \sum_{k=0}^{\infty} N_k, \tag{S-II.0.1}$$

$$E_{\mathrm{tot}} = \sum_{k=0}^{\infty} N_k \varepsilon_k. \tag{S-II.0.2}$$

On the other hand, the number of the configurations of the N_{tot} systems that satisfy Eqs. (S-II.0.1) and (S-II.0.2) is given by

$$N_{\mathrm{con}} = \frac{N_{\mathrm{tot}}!}{\prod_{k=0}^{\infty} N_k!}. \tag{S-II.0.3}$$

Then the most probable state in this setup is the configuration that maximizes Eq. (S-II.0.3) under Eqs. (S-II.0.1) and (S-II.0.2). To solve this maximization problem, we use the method of Lagrange multipliers; the Lagrange function becomes

$$\ln N_{\mathrm{con}} - \alpha \sum_{k=0}^{\infty} N_k - \beta \sum_{k=0}^{\infty} N_k \varepsilon_k. \tag{S-II.0.4}$$

For $N \gg 0$, Stirling's formula reads

$$N_{\mathrm{tot}}! \approx N_{\mathrm{tot}}(\ln N_{\mathrm{tot}} - 1). \tag{S-II.0.5}$$

Applying Eq. (S-II.0.5) to Eq. (S-II.0.4), we obtain

$$\ln N_{\mathrm{con}} - \alpha \sum_{k=0}^{\infty} N_k - \beta \sum_{k=0}^{\infty} N_k \varepsilon_k = \ln \frac{N_{\mathrm{tot}}!}{\prod_{k=0}^{\infty} N_k!} - \alpha \sum_{k=0}^{\infty} N_k - \beta \sum_{k=0}^{\infty} N_k \varepsilon_k \tag{S-II.0.6}$$

$$\approx \ln \frac{N_{\mathrm{tot}}(\ln N_{\mathrm{tot}} - 1)}{\prod_{k=0}^{\infty} N_k(\ln N_k - 1)} - \alpha \sum_{k=0}^{\infty} N_k - \beta \sum_{k=0}^{\infty} N_k \varepsilon_k. \tag{S-II.0.7}$$

Then the variational conditions obtained from Eq. (S-II.0.4) with respect to $\{N_k\}_{n=0}^{\infty}$ are, for $n = 0, 1, 2, \ldots$,

$$0 = -\delta N_k(\ln N_k + \alpha + \beta\varepsilon_k). \tag{S-II.0.8}$$

Then we obtain, for $n = 1, 2, \ldots, \infty$.

$$N_k = e^{-\alpha} e^{-\beta\varepsilon_k}. \tag{S-II.0.9}$$

Then, by introducing $p_k^{\mathrm{can}} \coloneqq \frac{N_k}{N}$, we have

$$p_k^{\mathrm{can}} = \frac{e^{-\beta\varepsilon_k}}{\mathcal{Z}_\beta}, \tag{S-II.0.10}$$

where

$$\mathcal{Z}_\beta \coloneqq \sum_{k=0}^{\infty} e^{-\beta\varepsilon_k}. \tag{S-II.0.11}$$

Eq. (S-II.0.10) is called the canonical distribution. Note that the canonical distribution is not the unique equilibrium state of a system that is attached to a heat bath at inverse temperature β. We also note that the quantum counterpart of Eq. (S-II.0.10), which we call the Gibbs state is given by

$$\hat{\rho}_\beta = \frac{1}{\mathcal{Z}_\beta} \sum_{k=0}^{\infty} e^{-\beta\varepsilon_k} |k\rangle\langle k|. \tag{S-II.0.12}$$

In Eq. (S-II.0.10), we have derived the canonical distribution from the energy spectrum, that is, the Hamiltonian. In QAVB, we use the argument in a reversed way; that is, we define the Hamiltonian of VB from a probability distribution that has parameters to be estimated:

$$\varepsilon_k = -\frac{1}{\beta}\ln p_k^{\mathrm{can}} - \frac{1}{\beta}\ln \mathcal{Z}_\beta. \tag{S-II.0.13}$$

Note that, in QAVB, we set $\beta = 1$ when we define the Hamiltonian. The quantum counterpart of Eq. (S-II.0.13) is

$$\hat{H} = \sum_{k=0}^{\infty} \varepsilon_k |k\rangle\langle k|, \tag{S-II.0.14}$$

with Eq. (S-II.0.13).

S-III. GAUSSIAN MIXTURE MODEL

In the main paper, to demonstrate the performance of QAVB, we consider the estimation problem of the parameters and the number of clusters of the GMM studied in Refs. [3, 4]. The joint probability distribution of the GMM over an observable variable y_i and a hidden variable σ_i conditioned by a set of parameters θ is given by

$$p^{y,\sigma|\theta}(y_i, \sigma_i|\theta) = \sum_{k=1}^{K} \pi^k \mathcal{N}(y_i|\mu^k, (\Lambda^k)^{-1})\delta_{k,\sigma_i}, \tag{S-III.0.1}$$

where δ_{k,σ_i} is the Kronecker delta function, $\{\pi^k\}_{k=1}^K$ are the mixing coefficients of the GMM, and $\mathcal{N}(y_i|\mu^k, (\Lambda^k)^{-1})$ is a Gaussian distribution whose mean and precision, which is the inverse of its covariance, are μ^k and Λ^k, respectively [5]. Here, we have assumed that each hidden variable σ_i takes $1, \dots, K$; that is, $S^{\sigma_i} = \{k\}_{k=1}^K$ for each i. Note that, by using the one-hot notation [3, 4], we can construct an equivalent quantization scheme [6, 7]. To simplify the notation, we denote $\{\pi^k\}_{k=1}^K$, $\{\mu^k\}_{k=1}^K$, and $\{\Lambda^k\}_{k=1}^K$ by π, μ, and Λ, respectively, and we refer to $\{\pi, \mu, \Lambda\}$ collectively as θ.

Taking the logarithm of Eq. (S-III.0.1), we define the Hamiltonian of the GMM for σ_i with $y_i = y_i^{\mathrm{obs}}$ as

$$H_{\mathrm{cl}}^{\sigma_i|\theta} = -\ln p^{y,\sigma|\theta}(y_i^{\mathrm{obs}}, \sigma_i|\theta). \tag{S-III.0.2}$$

Then the Hamiltonian of the GMM for $\Sigma = \{\sigma_i\}_{i=1}^N$ with $Y = Y^{\mathrm{obs}}$ is given by $H_{\mathrm{cl}}^{\Sigma|\theta} = \sum_{i=1}^N H_{\mathrm{cl}}^{\sigma_i|\theta}$. Using a classical Hamiltonian $\hat{H}_{\mathrm{cl}}^{\Sigma|\theta}$, we can also define the quantum representation of $H_{\mathrm{cl}}^{\Sigma|\theta}$.

To introduce quantum fluctuations into $\hat{H}_{\mathrm{cl}}^{\Sigma|\theta}$, we add a noncommutative term $\hat{H}_{\mathrm{qu}}^{\Sigma} = \sum_{i=1}^N \hat{H}_{\mathrm{qu}}^{\sigma_i}$ that satisfies $[\hat{H}_{\mathrm{cl}}^{\Sigma|\theta}, \hat{H}_{\mathrm{qu}}^{\Sigma}] \neq 0$. In this paper, we adopt

$$\hat{H}_{\mathrm{qu}}^{\sigma_i} = \left(\bigotimes_{j=1}^{i-1} \hat{I}^{\sigma_j}\right) \otimes \left(\sum_{\substack{k=1,\dots,K,\\ l=k\pm1}} |\sigma_i = l\rangle\langle\sigma_i = k|\right) \otimes \left(\bigotimes_{j=i+1}^{N} \hat{I}^{\sigma_j}\right) \otimes \hat{I}^{\theta}, \tag{S-III.0.3}$$

where $|\sigma_i = 0\rangle = |\sigma_i = K\rangle$, and $|\sigma_i = K+1\rangle = |\sigma_i = 1\rangle$. We note that the form of $\hat{H}_{\mathrm{qu}}^{\sigma_i}$ is not limited to the above definition and has arbitrariness in general.

S-IV. PRIOR AND POSTERIOR DISTRIBUTIONS OF THE GMM

In Sec. S-III, we explained the GMM and its Hamiltonian. From the viewpoint of Bayes inference, prior and posterior distributions are quite important. Thus, we review those of the GMM here.

A. Prior distributions of the GMM

Conjugate prior distributions of a model yield posterior distributions that have the same functional forms with them. Thus, we often use conjugate prior distributions as prior distributions in VB, and, in this case, we can transform the

update rules of distributions of parameters into the update rules of hyperparameters, which characterize the conjugate prior distribution and the associated posterior distribution.

This section is devoted to introducing the conjugate prior distributions of the GMM. The explanation is divided into two since the prior distribution of the mixture coefficients π can be described independently from the other parameters, and the mean μ and precision Λ depend on each other.

1. *Prior distribution of* π

Let us denote, by $p^{\pi}_{\rm pr}(\pi)$, the prior distribution of π. The prior distribution of the mixture coefficients π is given by the Dirichlet distribution:

$$p^{\pi}_{\rm pr}(\pi) = \mathcal{D}(\pi|\alpha_{\rm pr}), \tag{S-IV.1.1}$$

where $\alpha_{\rm pr} = \{\alpha^k_{\rm pr}\}_{k=1}^K$, $C(\alpha_{\rm pr})$ is the normalization constant, and the Dirichlet distribution reads

$$\mathcal{D}(\pi|\alpha) = C(\alpha)\prod_{k=1}^{K}\left(\pi^k\right)^{\alpha^k-1}, \tag{S-IV.1.2}$$

where $\alpha = \{\alpha^k\}_{k=1}^K$. We will see that, in this case, the posterior distribution becomes the Dirichlet distribution again.

2. *Prior distributions of* μ *and* Λ

Next, we turn our attention to the prior distribution of μ and Λ. The prior distribution of them can be expressed as the product of the prior distributions of μ and Λ:

$$p^{\mu,\Lambda}_{\rm pr}(\mu,\Lambda) = p^{\mu|\Lambda}_{\rm pr}(\mu|\Lambda)p^{\Lambda}_{\rm pr}(\Lambda) \tag{S-IV.1.3}$$

$$= \prod_{k=1}^{K} p^{\mu^k|\Lambda^k}_{\rm pr}(\mu^k|\Lambda^k)p^{\Lambda^k}_{\rm pr}(\Lambda^k), \tag{S-IV.1.4}$$

where

$$p^{\mu^k|\Lambda^k}_{\rm pr}(\mu^k|\Lambda^k) = \mathcal{N}(\mu^k|m^k_{\rm pr},(\gamma^k_{\rm pr}\Lambda^k)^{-1}), \tag{S-IV.1.5}$$

$$p^{\Lambda^k}_{\rm pr}(\Lambda^k) = \mathcal{W}(\Lambda^k|W^k_{\rm pr},\nu^k_{\rm pr}). \tag{S-IV.1.6}$$

Here, the Wishart distribution is defined as

$$\mathcal{W}(\Lambda^k|W^k,\nu^k) = B|\Lambda^k|^{(\nu^k-D-1)/2}\exp\left(-\frac{1}{2}{\rm Tr}\left[(W^k)^{-1}\Lambda^k\right]\right), \tag{S-IV.1.7}$$

with

$$B(W^k,\nu^k) = |W^k|^{-\nu/2}\left(2^{\nu D/2}\pi^{D(D-1)/4}\prod_{i=1}^{D}\Gamma\left(\frac{\nu^k+1-i}{2}\right)\right)^{-1}, \tag{S-IV.1.8}$$

and D is the number of dimensions. Note that the Wishart distribution is defined when $\nu^k > D-1$ for $k = 1,\ldots,K$. The Gamma function $\Gamma(z)$ in Eq. (S-IV.1.8) is given by

$$\Gamma(z) = \int_0^\infty du\, u^{z-1}e^{-u}, \tag{S-IV.1.9}$$

for ${\rm Re}[z] \geq 0$.

B. Posterior distribution of Σ in VB

Here, we explicitly write down the update rule of hidden variables $\Sigma = \{\sigma_i\}_{i=1}^N$ in the GMM. The posterior distribution of hidden variables $\Sigma = \{\sigma_i\}_{i=1}^N$ is, in general, updated as

$$q_{t+1}^{\Sigma}(\Sigma) \propto \exp\left(\int_{\theta \in S^\theta} d\theta \, q_{t+1}^{\theta}(\theta) \ln p^{Y,\Sigma,\theta}(Y,\Sigma,\theta) \right). \tag{S-IV.2.1}$$

To simplify expressions, we introduce

$$\mathbb{E}_{q^\theta(\theta)}[\dots] := \int_{\theta \in S^\theta} d\theta \, q^\theta(\theta)[\dots]. \tag{S-IV.2.2}$$

Then, Eq. (S-IV.2.1) for the GMM is written as

$$\ln q_{t+1}^{\sigma_i}(\sigma_i) = \mathbb{E}_{q_{t+1}^{\theta}(\theta)}\Big[\ln p^{y,\sigma,\theta}(y_i,\sigma_i,\theta) \Big] \tag{S-IV.2.3}$$

$$= \mathbb{E}_{q_{t+1}^{\pi}(\pi)}\left[\ln p^{\sigma|\pi}(\sigma_i|\pi)\right] + \mathbb{E}_{q_{t+1}^{\mu,\Lambda}(\mu,\Lambda)}\left[\ln p^{y|\sigma,\mu,\Lambda}(y_i|\sigma_i,\mu,\Lambda)\right] + \text{const.} \tag{S-IV.2.4}$$

By simple calculations, we also have

$$\ln q_{t+1}^{\sigma_i}(\sigma_i) = -\sum_{\sigma_i \in S^\sigma} \mathbb{E}_{q_{t+1}^{\theta}(\theta)}\Big[H_{\text{cl}}^{\sigma_i|\theta}\Big]\delta_{k,\sigma_i} + \text{const.}, \tag{S-IV.2.5}$$

where $H_{\text{cl}}^{\sigma_i=k} = -\ln \pi^k \mathcal{N}(y_i|\mu^k,(\Lambda^k)^{-1})$. Taking the exponential of both sides of Eq. (S-IV.2.5) and normalizing, we finally obtain the update rules of hidden variables in the GMM:

$$q_{t+1}^{\sigma_i}(\sigma_i) = \sum_{k=1}^{K} \delta_{k,\sigma_i} r_i^k, \tag{S-IV.2.6}$$

where

$$r_i^k = \frac{\exp\Big(-\mathbb{E}_{q_{t+1}^{\theta}(\theta)}\Big[H_{\text{cl}}^{\sigma_i=k|\theta}\Big]\Big)}{\sum_{\sigma_i \in S^\sigma} \exp\Big(-\mathbb{E}_{q_{t+1}^{\theta}(\theta)}\Big[H_{\text{cl}}^{\sigma_i|\theta}\Big]\Big)}, \tag{S-IV.2.7}$$

for $i = 1, 2, \dots, N$.

C. Posterior distributions of θ in VB

The update rule for the parameter θ is written as

$$q_{t+1}^{\theta}(\theta) \propto p_{\text{pr}}^{\theta}(\theta) \exp\left(\sum_{\Sigma \in S^\Sigma} q_t^{\Sigma}(\Sigma) \ln p^{Y,\Sigma|\theta}(Y,\Sigma|\theta) \right) \tag{S-IV.3.1}$$

$$= p_{\text{pr}}^{\theta}(\theta) \prod_{i=1}^{N} \exp\left(\sum_{\sigma_i \in S^\sigma} q_t^{\sigma_i}(\sigma_i) \ln p^{y,\sigma|\theta}(y_i,\sigma_i|\theta) \right). \tag{S-IV.3.2}$$

We will write down an explicit formula of the update rules of the GMM using Eq. (S-IV.3.2). For later convenience, we first define some statistics as follows:

$$N^k = \sum_{i=1}^{N} r_i^k, \tag{S-IV.3.3a}$$

$$\bar{y}^k = \frac{1}{N_k} \sum_{i=1}^{N} r_i^k y_i, \tag{S-IV.3.3b}$$

$$S^k = \frac{1}{N_k} \sum_{i=1}^{N} r_i^k (y_i - \bar{y}^k)(y_i - \bar{y}^k)^\top. \tag{S-IV.3.3c}$$

Using Eq. (S-IV.3.2), the posterior distribution of θ is computed as

$$\ln q_{t+1}^{\theta}(\pi,\mu,\Lambda) = \ln p_{\rm pr}^{\pi}(\pi) + \sum_{k=1}^{K}\Big[\ln p_{\rm pr}^{\mu^k|\Lambda^k}(\mu^k|\Lambda^k) + \ln p_{\rm pr}^{\Lambda^k}(\Lambda^k)\Big] + \sum_{i=1}^{N}\mathbb{E}_{q_t^{\sigma_i}(\sigma_i)}\left[\ln p^{\sigma|\pi}(\sigma_i|\pi)\right]$$
$$+ \sum_{i=1}^{N}\sum_{k=1}^{K}\mathbb{E}_{q_t^{\sigma_i}(\sigma_i)}\Big[\ln p^{y|\sigma,\pi,\mu,\Lambda}(y_i|\sigma_i,\pi,\mu,\Lambda)\Big] + \text{const.} \tag{S-IV.3.4}$$

Similarly to Eq. (S-IV.2.2), we have also defined

$$\mathbb{E}_{q^{\sigma_i}(\sigma_i)}[\dots] \coloneqq \sum_{\sigma_i\in S^{\sigma}} q^{\sigma_i}(\sigma_i)[\dots]. \tag{S-IV.3.5}$$

To go further, we decompose $p_{\rm pr}^{\theta}(\pi,\mu,\Lambda) = p_{\rm pr}^{\pi}(\pi)p^{\mu,\Lambda}(\mu,\Lambda)$ as in Sec. S-IV A, and show the details as follows.

1. *Posterior distribution of* π

We first consider the posterior distribution of π. From Eqs. (S-IV.1.1) and (S-IV.3.4), we obtain the posterior distributions as

$$\ln q_{t+1}^{\pi}(\pi) = \ln p_{\rm pr}^{\pi}(\pi) + \sum_{i=1}^{N}\mathbb{E}_{q_t^{\sigma_i}(\sigma_i)}\left[\ln p^{\sigma|\pi}(\sigma_i|\pi)\right] + \text{const.} \tag{S-IV.3.6}$$

$$= \sum_{k=1}^{K}(\alpha_{\rm pr}^{k}-1)\ln\pi^k + \sum_{k=1}^{K}\left(\sum_{i=1}^{N} r_i^k\right)\ln\pi^k + \text{const.} \tag{S-IV.3.7}$$

$$= \sum_{k=1}^{K}(\alpha_{\rm pr}^{k}-1)\ln\pi^k + \sum_{k=1}^{K} N^k\ln\pi^k + \text{const.}, \tag{S-IV.3.8}$$

in which we have used

$$\mathbb{E}_{q^{\sigma_i}(\sigma_i)}\Big[\delta_{k,\sigma_i}\Big] = r_i^k, \tag{S-IV.3.9}$$

$$\sum_{i=1}^{N}\mathbb{E}_{q^{\sigma_i}(\sigma_i)}\left[\ln p^{\sigma|\pi}(\sigma_i|\pi)\right] = \left(\sum_{i=1}^{N} r_i^k\right)\ln\pi^k \tag{S-IV.3.10}$$

$$= N^k\ln\pi^k. \tag{S-IV.3.11}$$

Then we get

$$q_{t+1}^{\pi}(\pi) = \mathcal{D}(\pi|\alpha), \tag{S-IV.3.12}$$

where $\alpha = \{\alpha^k\}_{k=1}^{K}$ and

$$\alpha^k = \alpha_{\rm pr}^{k} + N^k, \tag{S-IV.3.13}$$

for $k = 1, 2, \dots, K$. Note that Eq. (S-IV.3.12) has the same functional form with Eq. (S-IV.1.1). This property is important to simplify the calculations of VB.

2. *Posterior distributions of* μ *and* Λ

Next, we derive the update rules for posterior distributions of μ and Λ. From Eqs. (S-IV.1.5), (S-IV.1.6), and (S-IV.3.4), we obtain the posterior distributions as

$$\ln q_{t+1}^{\mu,\Lambda}(\mu,\Lambda)$$
$$= \sum_{k=1}^{K}\Big[\ln p_{\rm pr}^{\mu^k|\Lambda^k}(\mu^k|\Lambda^k) + \ln p_{\rm pr}^{\Lambda^k}(\Lambda^k)\Big] + \sum_{i=1}^{N}\mathbb{E}_{q_t^{\sigma_i}(\sigma_i)}\Big[\ln p^{y|\sigma,\pi,\mu,\Lambda}(y_i|\sigma_i,\pi,\mu,\Lambda)\Big] + \text{const.} \tag{S-IV.3.14}$$

$$= \sum_{k=1}^{K} \Big[\ln \mathcal{N}(\mu^k | m_{\rm pr}^k, (\gamma_{\rm pr}^k \Lambda^k)^{-1}) + \ln \mathcal{W}(\Lambda^k | W_{\rm pr}^k, \nu_{\rm pr}^k) \Big] + \sum_{i=1}^{N} \sum_{k=1}^{K} \mathbb{E}_{q_t^{\sigma_i}(\sigma_i)} \Big[\delta_{k,\sigma_i} \Big] \ln \mathcal{N}(y_i | \mu^k, (\Lambda^k)^{-1}) + \text{const.} \tag{S-IV.3.15}$$

$$= \sum_{k=1}^{K} \Big[\ln \mathcal{N}(\mu^k | m_{\rm pr}^k, (\gamma_{\rm pr}^k \Lambda^k)^{-1}) + \ln \mathcal{W}(\Lambda^k | W_{\rm pr}^k, \nu_{\rm pr}^k) \Big] + \sum_{i=1}^{N} \sum_{k=1}^{K} r_i^k \ln \mathcal{N}(y_i | \mu^k, (\Lambda^k)^{-1}) + \text{const.} \tag{S-IV.3.16}$$

Then, with simple calculations, we have the updated distribution over the parameters μ and Σ given by

$$q_{t+1}^{\mu^k|\Lambda^k}(\mu^k|\Lambda^k) = \mathcal{N}(\mu^k | m^k, (\gamma^k \Lambda^k)^{-1}), \tag{S-IV.3.17a}$$

$$q_{t+1}^{\Lambda^k}(\Lambda^k) = \mathcal{W}(\Lambda^k | W^k, \nu^k), \tag{S-IV.3.17b}$$

where

$$\gamma^k = \gamma_{\rm pr}^k + N^k, \tag{S-IV.3.18a}$$

$$m^k = \frac{1}{\gamma^k}(\gamma_{\rm pr}^k m_{\rm pr}^k + N^k \bar{y}^k), \tag{S-IV.3.18b}$$

$$(W^k)^{-1} = (W_{\rm pr}^k)^{-1} + N^k S^k + \frac{\gamma_{\rm pr}^k N^k}{\gamma^k}(\bar{y}^k - m_{\rm pr}^k)(\bar{y}^k - m_{\rm pr}^k)^\top, \tag{S-IV.3.18c}$$

$$\nu^k = \nu_{\rm pr}^k + N^k. \tag{S-IV.3.18d}$$

When we use the implementations of VB and QAVB described, we set $\nu_0^k = D - 1$ since $N^k > 0$ almost surely if initial distributions of Σ is properly initialized.

S-V. POSTERIOR DISTRIBUTION IN DAVB

In the previous section, we introduced the conjugate prior distribution and the associated posterior distribution of the GMM in the case of VB. Here, we consider the posterior distributions of the GMM in the case of DAVB.

A. Quick review of DAVB

We here try to briefly explain the key idea of DAVB [8]. In DAVB, we introduce thermal fluctuations into probability distributions by assuming the canonical distribution; then the total joint probability distribution becomes

$$p_{\rm SA}^{Y,\Sigma,\theta}(Y,\Sigma,\theta;\beta^{\rm pr},\beta) \coloneqq \Big[p_{\rm pr}^{\theta}(\theta)\Big]^{\beta^{\rm pr}} \Big[p^{Y,\Sigma|\theta}(Y,\Sigma|\theta)\Big]^{\beta}, \tag{S-V.1.1}$$

We stress that Eq. (S-V.1.1) has two inverse temperatures for the prior distribution and the GMM: $\beta^{\rm pr}$ and β. It will turn out to be reasonable to introduce two different inverse temperatures.

The update equations of Σ and θ for DAVB is then computed by minimizing the following KL divergence:

$$\mathrm{KL}\left(q^{\Sigma,\theta}(\Sigma,\theta) \middle\| p_{\rm SA}^{\Sigma,\theta|Y}(\Sigma,\theta|Y;\beta^{\rm pr},\beta)\right) \coloneqq -\sum_{\Sigma\in S^{\Sigma}} \int_{\theta\in S^{\theta}} d\theta\, q^{\Sigma,\theta}(\Sigma,\theta) \ln \frac{p_{\rm SA}^{\Sigma,\theta|Y}(\Sigma,\theta|Y;\beta^{\rm pr},\beta)}{q^{\Sigma,\theta}(\Sigma,\theta)}. \tag{S-V.1.2}$$

B. Posterior distribution of σ_i in DAVB

Following the same procedure employed in VB, we also obtain

$$\ln q_{t+1}^{\sigma_i}(\sigma_i) = -\beta \sum_{\sigma_i \in S^{\sigma}} \mathbb{E}_{q_{t+1}^{\theta}(\theta)}\Big[H_{\rm cl}^{\sigma_i|\theta}\Big]\delta_{k,\sigma_i} + \text{const.}, \tag{S-V.2.1}$$

where $H_{\rm cl}^{\sigma_i = k} = -\ln \pi^k \mathcal{N}(y_i|\mu^k, (\Lambda^k)^{-1})$. Taking the exponential of Eq. (S-V.2.1), we obtain

$$q_{t+1}^{\sigma_i}(\sigma_i) = \sum_{k=1}^{K} \delta_{k,\sigma_i} r_i^k, \tag{S-V.2.2}$$

where

$$r_i^k = \frac{\exp\Big(-\beta \mathbb{E}_{q^\theta_{t+1}(\theta)}\Big[H_{\text{cl}}^{\sigma_i=k|\theta}\Big]\Big)}{\sum_{\sigma_i\in S^\sigma}\exp\Big(-\beta \mathbb{E}_{q^\theta_{t+1}(\theta)}\Big[H_{\text{cl}}^{\sigma_i|\theta}\Big]\Big)}. \tag{S-V.2.3}$$

C. Posterior distribution of θ in DAVB

Next, we turn our attention to the update rules of θ in the case of DAVB.

1. Posterior distribution of π in DAVB

We first consider the update rule of π. The posterior distribution of π in the case of DAVB is computed as

$$\ln q^\pi_{t+1}(\pi) = \beta^{\text{pr}} \ln p^\pi_{\text{pr}}(\pi) + \beta \sum_{i=1}^N \mathbb{E}_{q_t^{\sigma_i}(\sigma_i)}\left[\ln p^{\sigma|\pi}(\sigma_i|\pi)\right] + \text{const.} \tag{S-V.3.1}$$

$$= \sum_{k=1}^K \beta^{\text{pr}}(\alpha^k_{\text{pr}}-1)\ln\pi^k + \beta\sum_{k=1}^K\left(\sum_{i=1}^N r_i^k\right)\ln\pi^k + \text{const.} \tag{S-V.3.2}$$

$$= \sum_{k=1}^K \beta^{\text{pr}}(\alpha^k_{\text{pr}}-1)\ln\pi^k + \beta\sum_{k=1}^K N^k\ln\pi^k + \text{const.} \tag{S-V.3.3}$$

$$= \sum_{k=1}^K \Big[\Big\{\beta^{\text{pr}}(\alpha^k_{\text{pr}}-1) + \beta N^k + 1\Big\} - 1\Big]\ln\pi^k + \text{const.} \tag{S-V.3.4}$$

Thus, Eq. (S-IV.3.13), which is the update rule of π in VB, becomes

$$\alpha^k = \beta^{\text{pr}}(\alpha^k_{\text{pr}}-1) + \beta N^k + 1. \tag{S-V.3.5}$$

2. Posterior distributions of μ and Σ in DAVB

Next, we compute the posterior distribution of μ and Λ in the case of DAVB:

$$\ln q^{\mu,\Lambda}_{t+1}(\mu,\Lambda)$$

$$= \beta^{\text{pr}}\sum_{k=1}^K\Big[\ln p^{\mu^k|\Lambda^k}_{\text{pr}}(\mu^k|\Lambda^k) + \ln p^{\Lambda^k}_{\text{pr}}(\Lambda^k)\Big] + \beta\sum_{i=1}^N \mathbb{E}_{q_t^{\sigma_i}(\sigma_i)}\Big[\ln p^{y|\sigma,\pi,\mu,\Lambda}(y_i|\sigma_i,\pi,\mu,\Lambda)\Big] + \text{const.} \tag{S-V.3.6}$$

$$= \beta^{\text{pr}}\sum_{k=1}^K\Big[\ln\mathcal{N}(\mu^k|m^k_{\text{pr}},(\gamma^k_{\text{pr}}\Lambda^k)^{-1}) + \ln\mathcal{W}(\Lambda^k|W^k_{\text{pr}},\nu^k_{\text{pr}})\Big] + \beta\sum_{i=1}^N\sum_{k=1}^K \mathbb{E}_{q_t^{\sigma_i}(\sigma_i)}\Big[\delta_{k,\sigma_i}\Big]\ln\mathcal{N}(y_i|\mu^k,(\Lambda^k)^{-1}) + \text{const.} \tag{S-V.3.7}$$

$$= \beta^{\text{pr}}\sum_{k=1}^K\Big[\ln\mathcal{N}(\mu^k|m^k_{\text{pr}},(\gamma^k_{\text{pr}}\Lambda^k)^{-1}) + \ln\mathcal{W}(\Lambda^k|W^k_{\text{pr}},\nu^k_{\text{pr}})\Big] + \beta\sum_{k=1}^K\left(\sum_{i=1}^N r_i^k\right)\ln\mathcal{N}(y_i|\mu^k,(\Lambda^k)^{-1}) + \text{const.} \tag{S-V.3.8}$$

$$= \beta^{\text{pr}}\sum_{k=1}^K\Big[\ln\mathcal{N}(\mu^k|m^k_{\text{pr}},(\gamma^k_{\text{pr}}\Lambda^k)^{-1}) + \ln\mathcal{W}(\Lambda^k|W^k_{\text{pr}},\nu^k_{\text{pr}})\Big] + \beta\sum_{k=1}^K N^k\ln\mathcal{N}(y_i|\mu^k,(\Lambda^k)^{-1}) + \text{const.} \tag{S-V.3.9}$$

Then the posterior distribution of μ^k conditioned by Λ^k is written as

$$q^{\mu^k|\Lambda^k}_{t+1}(\mu^k|\Lambda^k) = \mathcal{N}(\mu^k|m^k,(\gamma^k\Lambda^k)^{-1}), \tag{S-V.3.10}$$

where

$$\gamma^k = \beta^{\text{pr}}\gamma^k_{\text{pr}} + \beta N^k, \tag{S-V.3.11}$$

$$m^k = \frac{1}{\gamma^k}(\beta^{\rm pr}\gamma^k_{\rm pr}m^k_{\rm pr} + \beta N^k \bar{y}^k). \tag{S-V.3.12}$$

Thus, Eqs. (S-IV.3.18a) and (S-IV.3.18b) are transformed to Eqs. (S-V.3.11) and (S-V.3.12). Furthermore, Bayes' theorem leads to the following calculation:

$$\ln q^{\Lambda^k}_{t+1}(\Lambda^k) = \ln q^{\mu^k,\Lambda^k}_{t+1}(\mu^k,\Lambda^k) - \ln q^{\mu^k|\Lambda^k}_{t+1}(\mu^k|\Lambda^k) \tag{S-V.3.13}$$

$$= \beta^{\rm pr}\ln\mathcal{N}(\mu^k|m^k_{\rm pr},(\gamma^k_{\rm pr}\Lambda^k)^{-1}) + \beta^{\rm pr}\ln\mathcal{W}(\Lambda^k|W^k_{\rm pr},\nu^k_{\rm pr})$$
$$+ \beta\sum_{i=1}^{N} r^k_i \ln\mathcal{N}(y_i|\mu^k,(\Lambda^k)^{-1}) - \ln\mathcal{N}(\mu^k|m^k,(\gamma^k\Lambda^k)^{-1}) + \text{const.} \tag{S-V.3.14}$$

$$= \beta^{\rm pr}\frac{1}{2}\ln|\Lambda^k| - \beta^{\rm pr}\frac{1}{2}(\mu^k - m^k_{\rm pr})^\top(\gamma^k_0\Lambda^k)(\mu^k - m^k_{\rm pr}) + \beta^{\rm pr}\frac{1}{2}(\nu^k_{\rm pr} - D - 1)\ln|\Lambda^k| - \beta^{\rm pr}\frac{1}{2}\mathrm{Tr}[(W^k_{\rm pr})^{-1}\Lambda^k]$$
$$+ \beta N^k\frac{1}{2}\ln\Lambda^k - \beta\frac{1}{2}\sum_{i=1}^{N} r^k_i(y_i - \mu^k)\Lambda^k(y_i - \mu^k) - \frac{1}{2}\ln|\Lambda^k| + \frac{1}{2}(\mu^k - m^k)(\gamma^k\Lambda^k)(\mu^k - m^k) + \text{const.}, \tag{S-V.3.15}$$

Thus, the posterior distribution of Λ^k has the form

$$\ln q^{\Lambda^k}_{t+1}(\Lambda^k) = \frac{1}{2}(\nu^k - D - 1)\ln|\Lambda^k| - \frac{1}{2}\mathrm{Tr}[(W^k)^{-1}\Lambda^k], \tag{S-V.3.16}$$

where

$$(W^k)^{-1} = \beta^{\rm pr}\gamma^k_{\rm pr}(\mu^k - m^k_{\rm pr})(\mu^k - m^k_{\rm pr})^\top + \beta^{\rm pr}(W^k_{\rm pr})^{-1} + \beta\sum_{i=1}^{N} r^k_i(y_i - \mu^k)(y_i - \mu^k)^\top - \gamma^k(\mu^k - m^k)(\mu^k - m^k)^\top \tag{S-V.3.17a}$$

$$= \beta^{\rm pr}\gamma^k_{\rm pr}(\mu^k - m^k_{\rm pr})(\mu^k - m^k_{\rm pr})^\top + \beta^{\rm pr}(W^k_{\rm pr})^{-1}$$
$$+ \beta N^k S^k + \beta N^k(\bar{y}^k - \mu^k)(\bar{y} - \mu^k)^\top - \gamma^k(\mu^k - m^k)(\mu^k - m^k)^\top \tag{S-V.3.17b}$$

$$= \beta^{\rm pr}(W^k_{\rm pr})^{-1} + \beta N^k S^k + \frac{\beta^{\rm pr}\beta\gamma^k_{\rm pr}N^k}{\gamma^k}(\bar{y}^k - m^k_{\rm pr})(\bar{y}^k - m^k_{\rm pr})^\top, \tag{S-V.3.17c}$$

$$\nu^k = \beta^{\rm pr}\nu^k_{\rm pr} + \beta N^k + (1 - \beta^{\rm pr})D. \tag{S-V.3.17d}$$

We have succeeded in transforming Eqs. (S-IV.3.18c) and (S-IV.3.18d) into Eqs. (S-V.3.17c) and (S-V.3.17d).

S-VI. POSTERIOR DISTRIBUTION IN QAVB

In this section, we derive the posterior distributions in the generalized version of QAVB.

A. Posterior distribution of σ_i in QAVB

We have shown the update rule of σ_i of VB in Eq. (S-IV.2.5). In the case of the generalized version of QAVB, the update rule for σ_i becomes

$$\ln\hat{\rho}^{\sigma_i}_{t+1} = \mathrm{Tr}_\theta\left[\hat{\rho}^\theta_{t+1}\left\{-\beta s^{\rm cl}\hat{H}^{\sigma_i|\theta}_{\rm cl} - \beta s^\Sigma\hat{H}^{\sigma_i}_{\rm qu}\right\}\right] + \text{const.} \tag{S-VI.1.1}$$

where

$$\hat{H}^{\sigma_i|\theta}_{\rm cl} = \int_{\sigma_i\in S^\sigma} H^{\sigma_i|\theta}_{\rm cl}\hat{P}^{\sigma_i,\theta}, \tag{S-VI.1.2}$$

$$H^{\sigma_i=k}_{\rm cl} = -\ln\pi^k\mathcal{N}(y_i|\mu^k,(\Lambda^k)^{-1}), \tag{S-VI.1.3}$$

$\hat{P}^{\sigma_i,\theta} := |\sigma_i,\theta\rangle\langle\sigma_i,\theta|$, and $S^\sigma = \{k\}^K_{k=1}$. Taking the exponential of Eq. (S-VI.1.1), we obtain

$$\hat{\rho}^{\sigma_i}_{t+1} \propto \exp\left(\mathrm{Tr}_\theta\left[\hat{\rho}^\theta_{t+1}\left\{-\beta s^{\rm cl}\hat{H}^{\sigma_i|\theta}_{\rm cl} - \beta s^\Sigma\hat{H}^{\sigma_i}_{\rm qu}\right\}\right]\right), \tag{S-VI.1.4}$$

where the normalization condition is determined to satisfy the trace condition $\mathrm{Tr}_{\sigma_i}[\hat{\rho}^{\sigma_i}] = 1$.

B. Posterior distribution of θ in QAVB

Here, we write down the explicit formulas of posterior distributions of the GMM in the generalized version of QAVB. As explained in Sec. S-IV, the Hamiltonian of the conjugate prior distribution of the GMM can be decomposed as

$$H_{\rm pr}^{\theta} = H_{\rm pr}^{\pi} + H_{\rm pr}^{\mu|\Lambda} + H_{\rm pr}^{\Lambda}. \tag{S-VI.2.1}$$

Furthermore, due to the definition of the conjugate prior distributions, the Hamiltonians of the posterior distributions of QAVB have the same form

$$H_{\rm cl}^{\theta} = H_{\rm cl}^{\pi} + H_{\rm cl}^{\mu|\Lambda} + H_{\rm cl}^{\Lambda}. \tag{S-VI.2.2}$$

In this section, we describe the update rules using the above Hamiltonian formulations.

C. Definitions of Quantum states and operators of θ

For later convenience, we define the quantum states of $\theta = \{\pi, \mu, \Lambda\}$, π, μ, and Λ by $|\theta\rangle$, $|\pi\rangle$, $|\mu\rangle$, and $|\Lambda\rangle$ by $|\theta\rangle$, $|\pi\rangle$, $|\mu\rangle$, and $|\Lambda\rangle$, respectively. These ket vectors satisfy

$$|\theta\rangle = |\pi\rangle \otimes |\mu\rangle \otimes |\Lambda\rangle\,. \tag{S-VI.3.1}$$

Next, we define the projection operators of θ, π, μ, and Λ denoted by $\hat{P}^{\theta}$, $\hat{P}^{\pi}$, $\hat{P}^{\mu}$, $\hat{P}^{\Lambda}$, respectively:

$$\hat{P}^{\theta} = |\theta\rangle\langle\theta|\,, \tag{S-VI.3.2a}$$
$$\hat{P}^{\pi} = |\pi\rangle\langle\pi|\,, \tag{S-VI.3.2b}$$
$$\hat{P}^{\mu} = |\mu\rangle\langle\mu|\,, \tag{S-VI.3.2c}$$
$$\hat{P}^{\Lambda} = |\Lambda\rangle\langle\Lambda|\,. \tag{S-VI.3.2d}$$

Similarly, we also define the identity operators of θ, π, μ, and Λ by $\hat{I}^{\theta}$, $\hat{I}^{\pi}$, $\hat{I}^{\mu}$, $\hat{I}^{\Lambda}$, respectively:

$$\hat{I}^{\theta} = \int d\theta\, |\theta\rangle\langle\theta|\,, \tag{S-VI.3.3a}$$
$$\hat{I}^{\pi} = \int d\pi\, |\pi\rangle\langle\pi|\,, \tag{S-VI.3.3b}$$
$$\hat{I}^{\mu} = \int d\mu\, |\mu\rangle\langle\mu|\,, \tag{S-VI.3.3c}$$
$$\hat{I}^{\Lambda} = \int d\Lambda\, |\Lambda\rangle\langle\Lambda|\,. \tag{S-VI.3.3d}$$

We also denote, by $|\mu^k\rangle$ and $|\Lambda^k\rangle$, the quantum states of μ^k and Λ^k, respectively. Then the projection operators of μ^k and Λ^k are, respectively, given by

$$\hat{P}^{\mu_k} = |\mu_k\rangle\langle\mu_k|\,, \tag{S-VI.3.4a}$$
$$\hat{P}^{\Lambda_k} = |\Lambda_k\rangle\langle\Lambda_k|\,. \tag{S-VI.3.4b}$$

Furthermore, the identity operators of μ^k and Λ^k reads, respectively,

$$\hat{I}^{\mu} = \bigotimes_{k=1}^{K} \hat{I}^{\mu^k}, \tag{S-VI.3.5a}$$
$$\hat{I}^{\Lambda} = \bigotimes_{k=1}^{K} \hat{I}^{\Lambda^k}. \tag{S-VI.3.5b}$$

Note that the following equalities hold:

$$\hat{P}^{\mu} = \bigotimes_{k=1}^{K} \hat{P}^{\mu^k}, \tag{S-VI.3.6a}$$
$$\hat{P}^{\Lambda} = \bigotimes_{k=1}^{K} \hat{P}^{\Lambda^k}. \tag{S-VI.3.6b}$$

1. Posterior distribution of π in QAVB

The update equation of π of the GMM in the case of the generalized version of QAVB reads

$$\ln \hat{\rho}^{\pi}_{t+1} = -\hat{H}^{\pi}_{\mathrm{cl}} - \beta s^{\theta} \hat{H}^{\pi}_{\mathrm{qu}} + \mathrm{const.}, \tag{S-VI.3.7}$$

where

$$\hat{H}^{\pi}_{\mathrm{cl}} \coloneqq \int_{S^{\pi}} d\pi\, H^{\pi}_{\mathrm{cl}} \hat{P}^{\Sigma,\theta}, \tag{S-VI.3.8a}$$

$$H^{\pi}_{\mathrm{cl}} \coloneqq -\ln\Big(\mathcal{D}(\pi|\alpha)\Big), \tag{S-VI.3.8b}$$

$$\alpha^{k} \coloneqq \beta^{\mathrm{pr}}(\alpha^{k}_{\mathrm{pr}} - 1) + \beta s^{\mathrm{cl}} N^{k} + 1. \tag{S-VI.3.8c}$$

We impose the commutation relation on $\hat{H}^{\pi}_{\mathrm{qu}}$, $[\hat{H}^{\pi}_{\mathrm{qu}}, \hat{I}^{\Sigma} \otimes \hat{\pi} \otimes \hat{I}^{\mu} \otimes \hat{I}^{\Lambda}] \neq 0$. Here, $\hat{P}^{\pi} = |\pi\rangle\langle\pi|$, $\hat{\pi} = \int_{\pi \in S^{\pi}} d\pi\, \pi \hat{P}^{\pi}$, and S^{π} is the domain of π. Taking the exponential of Eq. (S-VI.3.7), we obtain

$$\hat{\rho}^{\pi}_{t+1} \propto \exp\Big(- \hat{H}^{\pi}_{\mathrm{cl}} - \beta s^{\theta} \hat{H}^{\pi}_{\mathrm{qu}}\Big). \tag{S-VI.3.9}$$

2. Posterior distributions of μ and Σ in QAVB

Similarly, the update rules of μ is written as

$$\ln \hat{\rho}^{\mu^{k}|\Lambda^{k}}_{t+1} = -\hat{H}^{\mu^{k}|\Lambda^{k}}_{\mathrm{cl}} - \beta s^{\theta} \hat{H}^{\mu^{k}}_{\mathrm{qu}} + \mathrm{const.}, \tag{S-VI.3.10}$$

where

$$\hat{H}^{\mu^{k}|\Lambda^{k}}_{\mathrm{cl}} \coloneqq \int_{\mu^{k} \in S^{\mu^{k}}} d\mu^{k} \int_{\Lambda^{k} \in S^{\Lambda^{k}}} d\Lambda^{k}\, H^{\mu^{k}|\Lambda^{k}}_{\mathrm{cl}} \hat{P}^{\Sigma,\theta}, \tag{S-VI.3.11a}$$

$$H^{\mu^{k}|\Lambda^{k}}_{\mathrm{cl}} \coloneqq -\ln\Big(\mathcal{N}(\mu^{k}|m^{k}, (\gamma^{k}\Lambda^{k})^{-1})\Big), \tag{S-VI.3.11b}$$

$$\gamma^{k} \coloneqq \beta^{\mathrm{pr}} \gamma^{k}_{\mathrm{pr}} + \beta s^{\mathrm{cl}} N^{k},, \tag{S-VI.3.11c}$$

$$m^{k} \coloneqq \frac{1}{\gamma^{k}}(\beta^{\mathrm{pr}} \gamma^{k}_{\mathrm{pr}} m^{k}_{\mathrm{pr}} + \beta s^{\mathrm{cl}} N^{k} \bar{y}^{k}). \tag{S-VI.3.11d}$$

We impose the commutation relation on $\hat{H}^{\mu^{k}}_{\mathrm{qu}}$:

$$\left[\hat{H}^{\mu^{k}}_{\mathrm{qu}}, \hat{I}^{\Sigma} \otimes \hat{I}^{\pi} \otimes \left(\bigotimes_{j=1}^{k-1} \hat{I}^{\mu^{j}}\right) \otimes \hat{\mu}^{k} \otimes \left(\bigotimes_{j=k+1}^{K} \hat{I}^{\mu^{j}}\right) \otimes \hat{I}^{\Lambda}\right] \neq 0. \tag{S-VI.3.12}$$

Here, $\hat{P}^{\mu^{k},\Lambda^{k}} \coloneqq \left|\mu^{k}, \Lambda^{k}\right\rangle \left\langle \mu^{k}, \Lambda^{k}\right|$, with $\hat{\mu}^{k} \coloneqq \int_{\mu^{k} \in S^{\mu^{k}}} d\mu^{k}\, \mu^{k} \hat{P}^{\mu^{k}}$ with $\hat{P}^{\mu^{k}} \coloneqq \left|\mu^{k}\right\rangle \left\langle \mu^{k}\right|$, and $S^{\mu^{k}}$ and $S^{\Lambda^{k}}$ are the domains of μ^{k} and Λ^{k}, respectively. Taking the exponential of Eq. (S-VI.3.10), we obtain

$$\hat{\rho}^{\mu^{k}|\Lambda^{k}}_{t+1} \propto \exp\Big(- \hat{H}^{\mu^{k}|\Lambda^{k}}_{\mathrm{cl}} - \beta s^{\theta} \hat{H}^{\mu^{k}}_{\mathrm{qu}}\Big). \tag{S-VI.3.13}$$

The update rule of Λ is written as

$$\ln \hat{\rho}^{\Lambda^{k}}_{t+1} = -\hat{H}^{\Lambda^{k}}_{\mathrm{cl}} - \beta s^{\theta} \hat{H}^{\Lambda^{k}}_{\mathrm{qu}} + \mathrm{const.}, \tag{S-VI.3.14}$$

where

$$\hat{H}^{\Lambda^{k}}_{\mathrm{cl}} \coloneqq \int_{\Lambda^{k} \in S^{\Lambda^{k}}} d\Lambda^{k}\, H^{\Lambda^{k}}_{\mathrm{cl}} \hat{P}^{\Sigma,\theta}, \tag{S-VI.3.15a}$$

$$H^{\Lambda^{k}}_{\mathrm{cl}} \coloneqq -\ln\Big(\mathcal{W}(\Lambda^{k}|W^{k}, \nu^{k})\Big) \tag{S-VI.3.15b}$$

$$W^{k} \coloneqq \beta^{\mathrm{pr}}(W^{k}_{\mathrm{pr}})^{-1} + \beta s^{\mathrm{cl}} N^{k} S^{k} + \frac{\beta^{\mathrm{pr}} \beta s^{\mathrm{cl}} \gamma^{k}_{\mathrm{pr}} N^{k}}{\gamma^{k}} (\bar{y}^{k} - m^{k}_{\mathrm{pr}})(\bar{y}^{k} - m^{k}_{\mathrm{pr}})^{\top}, \tag{S-VI.3.15c}$$

$$\nu^k := \beta^{\rm pr}\nu^k_{\rm pr} + \beta s^{\rm cl} N^k + (1-\beta^{\rm pr})D. \tag{S-VI.3.15d}$$

Here $\hat{P}^{\Lambda^k} := |\Lambda^k\rangle\langle\Lambda^k|$, $\hat{\Lambda}^k := \int_{\Lambda^k\in S^{\Lambda^k}} d\Lambda^k\,\Lambda^k\hat{P}^{\Lambda^k}$, S^{Λ^k} is the domain of Λ^k, and $\hat{H}^{\Lambda^k}_{\rm qu}$ is a Hamiltonian that satisfies

$$\left[\hat{H}^{\mu^k}_{\rm qu}, \hat{I}^{\Sigma}\otimes\hat{I}^{\pi}\otimes\hat{I}^{\mu}\otimes\left(\bigotimes_{j=1}^{k-1}\hat{I}^{\Lambda^j}\right)\otimes\hat{\Lambda}^k\otimes\left(\bigotimes_{j=k+1}^{K}\hat{I}^{\Lambda^j}\right)\right]\neq 0. \tag{S-VI.3.16}$$

Taking the exponential of Eq. (S-VI.3.14), we obtain

$$\hat{\rho}^{\Lambda^k}_{t+1} \propto \exp\Big(-\hat{H}^{\Lambda^k}_{\rm cl} - \beta s^{\theta}\hat{H}^{\Lambda^k}_{\rm qu}\Big). \tag{S-VI.3.17}$$

S-VII. QUANTIZATION OF θ IN QAVB

So far we have quantized Σ and now we explain the generalized version of QAVB and address the reason why we do not use it.

A. Generalized version of QAVB

By adding $\hat{H}^{\theta}_{\rm qu}$ that satisfies $[\hat{H}^{\theta}_{\rm qu}, \hat{\theta}]\neq 0$ to Eq. (13) and introducing the inverse temperature for the prior Hamiltonian, we obtain the generalized Gibbs operator given by

$$\hat{g}(\beta^{\rm pr},\beta,s^{\rm cl},s^{\Sigma},s^{\theta}) := \exp\Big(-\beta^{\rm pr}\hat{H}^{\theta}_{\rm pr} - \beta\big(s^{\rm cl}\hat{H}^{\Sigma|\theta}_{\rm cl} + s^{\Sigma}\hat{H}^{\Sigma}_{\rm qu} + s^{\theta}\hat{H}^{\theta}_{\rm qu}\big)\Big). \tag{S-VII.1.1}$$

In Eq. (S-VII.1.1), θ is also quantized in addition to Σ since the noncommutative term on θ is added. If we make use of Eq. (S-VII.1.1) instead of Eq. (13), we can formulate the fully quantized version of QAVB. Note that Eqs. (13) and (S-VII.1.1) are identical when $s^{\rm cl} = 1-s$, $s^{\Sigma} = s$, and $s^{\theta} = 0$.

If we use Eq. (S-VII.1.1) instead of Eq. (13) in QAVB, we can obtain the update equations of the fully quantized version of QAVB as follows. The derivation of the update equations is unchanged whether or not θ is quantized; then the update equations for the operator are written as

$$\hat{\rho}^{\Sigma}_{t+1} \propto \exp\Big({\rm Tr}_{\theta}\Big[\Big(\hat{I}^{\Sigma}\otimes\hat{\rho}^{\theta}_{t+1}\Big)\ln\hat{g}(\beta_t,s^{\rm cl}_t,s^{\Sigma}_t,s^{\theta}_t)\Big]\Big), \tag{S-VII.1.2}$$

$$\hat{\rho}^{\theta}_{t+1} \propto \exp\Big({\rm Tr}_{\Sigma}\Big[\Big(\hat{\rho}^{\Sigma}_t\otimes\hat{I}^{\theta}\Big)\ln\hat{g}(\beta_t,s^{\rm cl}_t,s^{\Sigma}_t,s^{\theta}_t)\Big]\Big). \tag{S-VII.1.3}$$

Here we note that the numerical cost still scales linearly with the number of data points N. In this paper, we call this algorithm the generalized version of QAVB.

B. Why we do not consider the generalized version of QAVB

In QAVB, we choose $\beta^{\rm pr} = 1$, $s^{\rm cl} = 1-s$, $s^{\Sigma} = s$, and $s^{\theta} = 0$. In this subsection, we state the reasons why we adopted $\beta^{\rm pr} = 1$ and $s^{\theta} = 0$. First, if we set $\beta^{\rm pr} > 1$, the posterior distribution may break the necessary condition of the Wishart distribution in the case of the GMM. That is the Wishart distribution can no longer act as a conjugate prior. Thus, we set $\beta^{\rm pr} = 1$. Second, when we set $s^{\theta}\neq 0$, we have to numerically solve the quantum Hamiltonian of parameters because we do not know the prior distributions associated with the quantum Hamiltonian of parameters and its computational cost is considered to be huge.

We have stated the reasons why we limited ourselves to the case of $\beta^{\rm pr} = 1$ and $s^{\theta} = 0$. Fortunately, QAVB with $\beta^{\rm pr} = 1$ and $s^{\theta} = 0$ works well; so, we do not need to consider the general version of QAVB.

S-VIII. QAVB IN THE PATH INTEGRAL FORMULATION

In general, quantum mechanics can also be formulated via the path integral formulation proposed by Feynman [9]. This section aims to reformulate QAVB by using the path integral formulation.

A. The quantum relative entropy and update equations of QAVB in the path integral formulation

So far, we have formulated QAVB in the operator formulation. On the other hand, there exists another formulation for quantum mechanics, that is, Feynman's path integral formulation [9–12]. Here, we propose the path integral formulation of QAVB for a better understanding and a prerequisite of to compare with the work of Sato *et al.* in Refs. [13, 14].

Using a variational function defined on paths $\{\Sigma_j\}_{j=1}^M$ and $\{\theta_j\}_{j=1}^M$,

$$\tilde{q}^{\Sigma,\theta}(\{\Sigma_j\}_{j=1}^M,\{\theta_j\}_{j=1}^M) \coloneqq \prod_{j=1}^M \left\langle \Sigma_j,\theta_j \left| \left[\hat{\rho}^{\Sigma,\theta}\right]^{\frac{1}{M}} \right| \Sigma_{j-1},\theta_{j-1} \right\rangle, \tag{S-VIII.1.1}$$

with $\Sigma_0 = \Sigma_M$ and $\theta_0 = \theta_M$, we define the KL divergence on paths to be minimized in the path integral formulation of QAVB as follows:

$$\begin{aligned}
&\widetilde{\mathrm{KL}}\left(\tilde{q}^{\Sigma,\theta}(\{\Sigma_j\}_{j=1}^M,\{\theta_j\}_{j=1}^M)\middle\| \frac{\tilde{g}(\{\Sigma_j\}_{j=1}^M,\{\theta_j\}_{j=1}^M;\beta^{\mathrm{pr}},\beta,s^{\mathrm{cl}},s^{\Sigma},s^{\theta})}{\tilde{\mathcal{Z}}(\beta^{\mathrm{pr}},\beta,s^{\mathrm{cl}},s^{\Sigma},s^{\theta})}\right)\\
&\coloneqq -\sum_{\Sigma_1,\dots,\Sigma_M\in S^{\Sigma}}\left[\prod_{j=1}^M\int_{\theta_j\in S^{\theta}} d\theta_j\right]\tilde{q}^{\Sigma,\theta}(\{\Sigma_j\}_{j=1}^M,\{\theta_j\}_{j=1}^M)\\
&\times\left[\ln\tilde{q}^{\Sigma,\theta}(\{\Sigma_j\}_{j=1}^M,\{\theta_j\}_{j=1}^M) - \ln\frac{\tilde{g}(\{\Sigma_j\}_{j=1}^M,\{\theta_j\}_{j=1}^M;\beta^{\mathrm{pr}},\beta,s^{\mathrm{cl}},s^{\Sigma},s^{\theta})}{\tilde{\mathcal{Z}}(\beta^{\mathrm{pr}},\beta,s^{\mathrm{cl}},s^{\Sigma},s^{\theta})}\right].
\end{aligned} \tag{S-VIII.1.2}$$

Here, we have used

$$\begin{aligned}
&\tilde{g}(\{\Sigma_j\}_{j=1}^M,\{\theta_j\}_{j=1}^M;\beta^{\mathrm{pr}},\beta,s^{\mathrm{cl}},s^{\Sigma},s^{\theta})\\
&\coloneqq \prod_{j=1}^M\left\langle\Sigma_j,\theta_j\left|\left[e^{\frac{1}{M}\hat{K}_{\mathrm{cl}}^{\Sigma,\theta}(\beta^{\mathrm{pr}},\beta,s^{\mathrm{cl}})}e^{\frac{-\beta}{M}s^{\Sigma}\hat{H}_{\mathrm{qu}}^{\Sigma}}e^{\frac{-\beta}{M}s^{\theta}\hat{H}_{\mathrm{qu}}^{\theta}}\right]\right|\Sigma_{j-1},\theta_{j-1}\right\rangle,
\end{aligned} \tag{S-VIII.1.3}$$

where

$$\hat{K}_{\mathrm{cl}}^{\Sigma,\theta}(\beta^{\mathrm{pr}},\beta,s^{\mathrm{cl}}) \coloneqq -\beta^{\mathrm{pr}}\hat{H}_{\mathrm{pr}}^{\theta} - \beta s^{\mathrm{cl}}\hat{H}_{\mathrm{cl}}^{\Sigma|\theta}, \tag{S-VIII.1.4}$$

and

$$\tilde{\mathcal{Z}}(\beta^{\mathrm{pr}},\beta,s^{\mathrm{cl}},s^{\Sigma},s^{\theta}) \coloneqq -\sum_{\Sigma_1,\dots,\Sigma_M\in S^{\Sigma}}\left[\prod_{j=1}^M\int_{\theta_j\in S^{\theta}}d\theta_j\right]\tilde{g}(\{\Sigma_j\}_{j=1}^M,\{\theta_j\}_{j=1}^M;\beta^{\mathrm{pr}},\beta,s^{\mathrm{cl}},s^{\Sigma},s^{\theta}), \tag{S-VIII.1.5}$$

where $|\Sigma_0,\theta_0\rangle = |\Sigma_M,\theta_M\rangle$ and M represents the number of beads in the Trotter dimension.

Here we mention a property of the partition function in the path integral formulation, Eq. (S-VIII.1.5). From Eq. (S-VII.1.1), the exact partition function is computed as

$$\mathcal{Z}(\beta^{\mathrm{pr}},\beta,s^{\mathrm{cl}},s^{\Sigma},s^{\theta}) \coloneqq \mathrm{Tr}_{\Sigma,\theta}[\hat{g}(\beta^{\mathrm{pr}},\beta,s^{\mathrm{cl}},s^{\Sigma},s^{\theta})]. \tag{S-VIII.1.6}$$

Equation (S-VIII.1.5) is an approximate form of the partition function and converges to the exact partition function with M infinite. That is, in the limit $M\to\infty$, Eq. (S-VIII.1.5) leads to Eq. (S-VIII.1.6):

$$\mathcal{Z}(\beta^{\mathrm{pr}},\beta,s^{\mathrm{cl}},s^{\Sigma},s^{\theta}) = \lim_{M\to\infty}\tilde{\mathcal{Z}}(\beta^{\mathrm{pr}},\beta,s^{\mathrm{cl}},s^{\Sigma},s^{\theta}). \tag{S-VIII.1.7}$$

As explained in the previous section, we rewrite minimization of Eq. (S-VIII.1.2) with the mean-field approximation as follows. Taking the logarithm of Eq. (S-VIII.1.5), we define the free energy function in the path integral formulation as

$$\tilde{\mathcal{F}}(\beta^{\mathrm{pr}},\beta,s^{\mathrm{cl}},s^{\Sigma},s^{\theta}) \coloneqq \ln\tilde{\mathcal{Z}}(\beta^{\mathrm{pr}},\beta,s^{\mathrm{cl}},s^{\Sigma},s^{\theta}). \tag{S-VIII.1.8}$$

Note that Eq. (S-VIII.1.8) is a constant. Next, we employ the mean-field approximation. In the mean-field approximation of the path integral formulation, we assume a mean-field variational function $\tilde{q}^{\Sigma,\theta}(\{\Sigma_j\}_{j=1}^M,\{\theta_j\}_{j=1}^M) = \tilde{q}^{\Sigma}(\{\Sigma_j\}_{j=1}^M)\tilde{q}^{\theta}(\{\theta_j\}_{j=1}^M)$. Thus the free energy function (S-VIII.1.8) can be decomposed into two parts:

$$\tilde{\mathcal{F}}(\beta^{\mathrm{pr}},\beta,s^{\mathrm{cl}},s^{\Sigma},s^{\theta}) = \tilde{\mathcal{G}}\left(\tilde{q}^{\Sigma}(\{\Sigma_j\}_{j=1}^M)\tilde{q}^{\theta}(\{\theta_j\}_{j=1}^M)\right)$$

$$+\widetilde{\mathrm{KL}}\left(\tilde{q}^{\Sigma}(\{\Sigma_j\}_{j=1}^{M})\tilde{q}^{\theta}(\{\theta_j\}_{j=1}^{M})\middle\|\frac{\tilde{g}(\{\Sigma_j\}_{j=1}^{M},\{\theta_j\}_{j=1}^{M};\beta^{\mathrm{pr}},\beta,s^{\mathrm{cl}},s^{\Sigma},s^{\theta})}{\tilde{\mathcal{Z}}(\beta^{\mathrm{pr}},\beta,s^{\mathrm{cl}},s^{\Sigma},s^{\theta})}\right), \tag{S-VIII.1.9}$$

with

$$\tilde{\mathcal{G}}\Big(\tilde{q}^{\Sigma}(\{\Sigma_j\}_{j=1}^{M})\tilde{q}^{\theta}(\{\theta_j\}_{j=1}^{M})\Big) \coloneqq \tilde{\mathcal{G}}^{\mathrm{cl}}\Big(\tilde{q}^{\Sigma}(\{\Sigma_j\}_{j=1}^{M})\tilde{q}^{\theta}(\{\theta_j\}_{j=1}^{M})\Big)+\tilde{\mathcal{G}}^{\Sigma}\Big(\tilde{q}^{\Sigma}(\{\Sigma_j\}_{j=1}^{M})\Big)+\tilde{\mathcal{G}}^{\theta}\Big(\tilde{q}^{\theta}(\{\theta_j\}_{j=1}^{M})\Big), \tag{S-VIII.1.10}$$

where

$$\tilde{\mathcal{G}}^{\mathrm{cl}}\Big(\tilde{q}^{\Sigma}(\{\Sigma_j\}_{j=1}^{M})\tilde{q}^{\theta}(\{\theta_j\}_{j=1}^{M})\Big) \coloneqq \sum_{\Sigma_1,\ldots,\Sigma_M\in S^{\Sigma}}\left[\prod_{j=1}^{M}\int_{\theta_j\in S^{\theta}}d\theta_j\right]\tilde{q}^{\Sigma}(\{\Sigma_j\}_{j=1}^{M})\tilde{q}^{\theta}(\{\theta_j\}_{j=1}^{M}) \times\left(\sum_{j=1}^{M}\frac{1}{M}\left\langle\Sigma_j,\theta_j\middle|\hat{K}_{\mathrm{cl}}^{\Sigma,\theta}(\beta^{\mathrm{pr}},\beta,s^{\mathrm{cl}})\middle|\Sigma_j,\theta_j\right\rangle-\ln\tilde{q}^{\Sigma}(\{\Sigma_j\}_{j=1}^{M})-\ln\tilde{q}^{\theta}(\{\theta_j\}_{j=1}^{M})\right), \tag{S-VIII.1.11}$$

$$\tilde{\mathcal{G}}^{\Sigma}\Big(\tilde{q}^{\Sigma}(\{\Sigma_j\}_{j=1}^{M})\Big)\coloneqq\sum_{\Sigma_1,\ldots,\Sigma_M\in S^{\Sigma}}\tilde{q}^{\Sigma}(\{\Sigma_j\}_{j=1}^{M})\sum_{j=1}^{M}\ln\left\langle\Sigma_j\middle|\left[e^{\frac{-\beta}{M}s^{\Sigma}\hat{H}_{\mathrm{qu}}^{\Sigma}}\right]\middle|\Sigma_{j-1}\right\rangle, \tag{S-VIII.1.12}$$

and

$$\tilde{\mathcal{G}}^{\theta}\Big(\tilde{q}^{\theta}(\{\theta_j\}_{j=1}^{M})\Big)\coloneqq\left[\prod_{j=1}^{M}\int_{\theta_j\in S^{\theta}}d\theta_j\right]\tilde{q}^{\theta}(\{\theta_j\}_{j=1}^{M})\sum_{j=1}^{M}\ln\left\langle\theta_j\middle|\left[e^{\frac{-\beta}{M}s^{\theta}\hat{H}_{\mathrm{qu}}^{\theta}}\right]\middle|\theta_{j-1}\right\rangle. \tag{S-VIII.1.13}$$

Note that minimization of Eq. (S-VIII.1.2) is identical to maximization of Eq. (S-VIII.1.10).

Then, taking the functional derivative of Eq. (S-VIII.1.2) with respect to $\tilde{q}^{\Sigma}(\{\Sigma_j\}_{j=1}^{M})$ and $\tilde{q}^{\theta}(\{\theta_j\}_{j=1}^{M})$, we obtain the update equations of QAVB in the path integral formulation,

$$\tilde{q}_{t+1}^{\Sigma}(\{\Sigma_j\}_{j=1}^{M}) \propto\exp\left(\left[\prod_{j=1}^{M}\int_{\theta_j\in S^{\theta}}d\theta_j\right]\tilde{q}_{t+1}^{\theta}(\{\theta_j\}_{j=1}^{M})\sum_{j=1}^{M}\frac{1}{M}\left\langle\Sigma_j,\theta_j\middle|\hat{K}_{\mathrm{cl}}^{\Sigma,\theta}(\beta^{\mathrm{pr}},\beta,s^{\mathrm{cl}})\middle|\Sigma_j,\theta_j\right\rangle\right) \times\prod_{j=1}^{M}\left\langle\Sigma_j\middle|\left[e^{\frac{-\beta}{M}s^{\Sigma}\hat{H}_{\mathrm{qu}}^{\Sigma}}\right]\middle|\Sigma_{j-1}\right\rangle, \tag{S-VIII.1.14}$$

and

$$\tilde{q}_{t+1}^{\theta}(\{\theta_j\}_{j=1}^{M}) \propto\exp\left(\sum_{\Sigma_1,\ldots,\Sigma_M\in S^{\Sigma}}\tilde{q}_{t}^{\Sigma}(\{\Sigma_j\}_{j=1}^{M})\sum_{j=1}^{M}\frac{1}{M}\left\langle\Sigma_j,\theta_j\middle|\hat{K}_{\mathrm{cl}}^{\Sigma,\theta}(\beta^{\mathrm{pr}},\beta,s^{\mathrm{cl}})\middle|\Sigma_j,\theta_j\right\rangle\right) \times\prod_{j=1}^{M}\left\langle\theta_j\middle|\left[e^{\frac{-\beta}{M}s^{\theta}\hat{H}_{\mathrm{qu}}^{\theta}}\right]\middle|\theta_{j-1}\right\rangle, \tag{S-VIII.1.15}$$

where t denotes the number of iterations, $\Sigma_0=\Sigma_M$ and $\theta_0=\theta_M$ are satisfied, and normalization constant is determined such that Eqs. (S-VIII.1.14) and (S-VIII.1.15) satisfy the condition of probability distributions on paths. In practical calculations, we can evaluate Eqs. (S-VIII.1.14) and (S-VIII.1.15) for any models by performing quantum Monte Carlo methods [? ? ?].

So far, we have derived the update equations of QAVB in the path integral formulation. In the end of this section, we show that update equations in the two formulations are identical. Namely, we prove that Eqs. (S-VIII.1.14) and (S-VIII.1.15) are equivalent to Eqs. (17) and (18) as follows. By relabeling the index j in $\{\theta_j\}_{j=1}^{M}$ and using $\left[\prod_{j=1}^{M-1}\int_{\theta_j\in S^{\theta}}d\theta_j\right]\tilde{q}_{t+1}^{\theta}(\{\theta_j\}_{j=1}^{M})=\left\langle\theta_M\middle|\hat{\rho}_{t+1}^{\theta}\middle|\theta_M\right\rangle$, Eq. (S-VIII.1.14) becomes

$$\tilde{q}_{t+1}^{\Sigma}(\{\Sigma_j\}_{j=1}^{M})$$

$$\propto \exp\left(\left[\prod_{j=1}^{M}\int_{\theta_j\in S^\theta} d\theta_j\right]\tilde{q}^{\theta}_{t+1}(\{\theta_j\}_{j=1}^M)\sum_{j=1}^{M}\frac{1}{M}\left\langle \Sigma_j,\theta_j\middle|\hat{K}^{\Sigma,\theta}_{\mathrm{cl}}(\beta^{\mathrm{pr}},\beta,s^{\mathrm{cl}})\middle|\Sigma_j,\theta_j\right\rangle\right)$$

$$\times\prod_{j=1}^{M}\left\langle\Sigma_j\middle|\left[e^{\frac{-\beta}{M}s^{\Sigma}\hat{H}^{\Sigma}_{\mathrm{qu}}}\right]\middle|\Sigma_{j-1}\right\rangle \tag{S-VIII.1.16}$$

$$= \exp\left(\int_{\theta_M\in S^\theta} d\theta_M\,\left\langle\theta_M\middle|\hat{\rho}^{\theta}_{t+1}\middle|\theta_M\right\rangle\sum_{j=1}^{M}\frac{1}{M}\left\langle \Sigma_j,\theta_j\middle|\hat{K}^{\Sigma,\theta}_{\mathrm{cl}}(\beta^{\mathrm{pr}},\beta,s^{\mathrm{cl}})\middle|\Sigma_j,\theta_j\right\rangle\right)$$

$$\times\prod_{j=1}^{M}\left\langle\Sigma_j\middle|\left[e^{\frac{-\beta}{M}s^{\theta}\hat{H}^{\Sigma}_{\mathrm{qu}}}\right]\middle|\Sigma_{j-1}\right\rangle. \tag{S-VIII.1.17}$$

We repeat the same calculation for Eq. (S-VIII.1.15):

$$\tilde{q}^{\theta}_{t+1}(\{\theta_j\}_{j=1}^M)$$

$$\propto \exp\left(\sum_{\Sigma_1,\ldots,\Sigma_M\in S^\Sigma}\tilde{q}^{\Sigma}_{t}(\{\Sigma_j\}_{j=1}^M)\sum_{j=1}^{M}\frac{1}{M}\left\langle \Sigma_j,\theta_j\middle|\hat{K}^{\Sigma,\theta}_{\mathrm{cl}}(\beta^{\mathrm{pr}},\beta,s^{\mathrm{cl}})\middle|\Sigma_j,\theta_j\right\rangle\right)$$

$$\times\prod_{j=1}^{M}\left\langle\theta_j\middle|\left[e^{\frac{-\beta}{M}s^{\theta}\hat{H}^{\theta}_{\mathrm{qu}}}\right]\middle|\theta_{j-1}\right\rangle \tag{S-VIII.1.18}$$

$$= \exp\left(\sum_{\Sigma_M\in S^\Sigma}\left\langle\Sigma_M\middle|\hat{\rho}^{\Sigma}_{t}\middle|\Sigma_M\right\rangle\sum_{j=1}^{M}\frac{1}{M}\left\langle \Sigma_j,\theta_j\middle|\hat{K}^{\Sigma,\theta}_{\mathrm{cl}}(\beta^{\mathrm{pr}},\beta,s^{\mathrm{cl}})\middle|\Sigma_j,\theta_j\right\rangle\right)$$

$$\times\prod_{j=1}^{M}\left\langle\theta_j\middle|\left[e^{\frac{-\beta}{M}s^{\theta}\hat{H}^{\theta}_{\mathrm{qu}}}\right]\middle|\theta_{j-1}\right\rangle. \tag{S-VIII.1.19}$$

Here, we have used $\sum_{\Sigma_1,\ldots,\Sigma_{M-1}\in S^\Sigma}\tilde{q}^{\Sigma}_{t}(\{\Sigma_j\}_{j=1}^M) = \left\langle\Sigma_M\middle|\hat{\rho}^{\Sigma}_{t}\middle|\Sigma_M\right\rangle$ from Eq. (S-VIII.1.18) to Eq. (S-VIII.1.19). Marginalizing $\Sigma_1,\ldots,\Sigma_{M-1}$ in Eq. (S-VIII.1.18) and $\theta_1,\ldots,\theta_{M-1}$ in Eq. (S-VIII.1.19) leads to Eqs. (S-VIII.1.14) and (S-VIII.1.15). Therefore, we conclude the update equations in the operator formulation and the path integral formulation are equivalent.

B. A prior attempt at designing a QA extension of VB using path integral formulation

An initial attempt was made in 2010 by Sato *et al.* [13, 14] to follow the path integral approach and create a QA extension of VB. However, the objective function used in Ref. [13, 14] does not equal the VB objective function in the classical limit. Thus, their algorithm does not minimize the VB objective function and is not a proper quantum extension of VB. In fact, in Refs. [2] the errors committed by Sato *et al.* [13, 14] have been pointed, and one correct way of deriving the updates that [13, 14] had intended has been formulated. On the other hand, the operator-based QAVB algorithm we analyze in this paper exactly corresponds to VB in the classical limit.

The main focus of this paper is to explain why and how the QAVB algorithm –based on the operator formulation of quantum mechanics – works and the main mechanisms for a quantum advantage. The dynamics of the correct path-integral based QAVB algorithms might be different from the QAVB analyzed in this paper, and we leave the analysis of the dynamics of such alternate algorithms as future work.

S-IX. APPLICATION TO REAL DATA

In this section, we apply QAVB to real data: (a) the anuran calls (MFCCs) dataset in the UCI repository [15, 16] and the MNIST dataset [17]. We set $s_0 = 1.0$, $\beta_0 = 10.0$, $\tau_1 = 450$, and $\tau_2 = 500$. We also put $\nu^k_{\mathrm{pr}} = 25.0$ and $\nu^k_{\mathrm{pr}} = 784.0$ for the datasets, respectively. In Fig. S-1, we plot the posterior log-likelihood values with the estimated numbers of clusters. These figures support our statements in the main text though the best solution of VB is slightly better than that of QAVB. Note that similar calculations are also shown in Ref. [2].

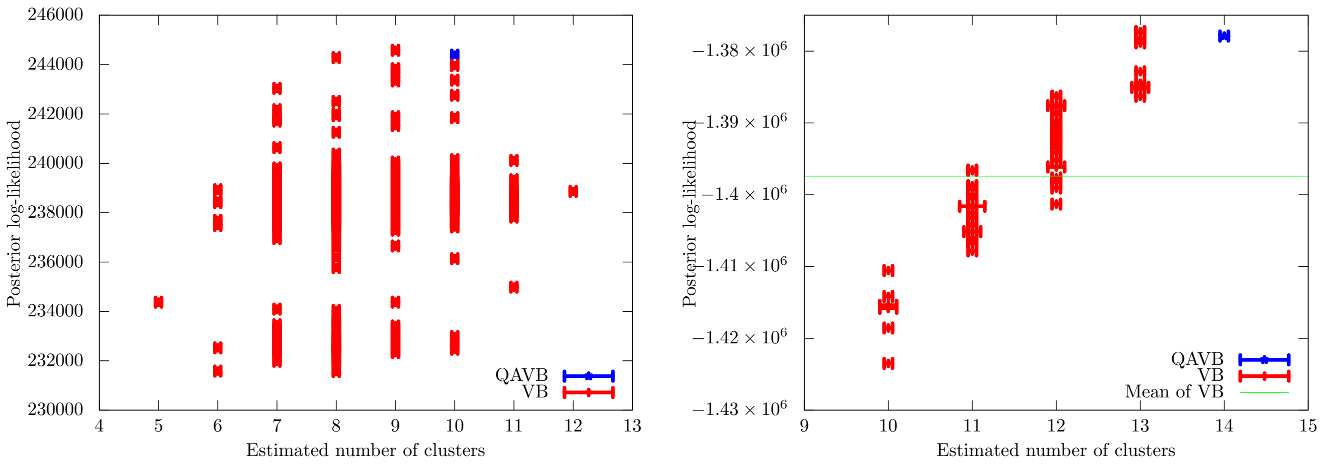

FIG. S-1. (a) the anuran calls (MFCCs) dataset in the UCI repository and (b) the MNIST dataset.

[1] H. Miyahara, K. Tsumura, and Y. Sughiyama, Journal of Statistical Mechanics: Theory and Experiment **2017**, 113404 (2017).
[2] H. Miyahara and Y. Sughiyama, Physical Review A **98**, 022330 (2018).
[3] C. M. Bishop, *Pattern recognition and machine learning* (springer, 2006).
[4] K. P. Murphy, *Machine learning: a probabilistic perspective* (MIT press, 2012).
[5] We have not gone into detail about the prior distribution of the GMM $p^{\theta}_{\mathrm{pr}}(\theta)$ because we do not quantize it in this paper. See Refs. [3, 4] if the reader is not familiar with it.
[6] H. Miyahara, K. Aihara, and W. Lechner, Physical Review A **101**, 012326 (2020).
[7] H. Miyahara and K. Tsumura, in *American Control Conference (ACC), 2016* (2016).
[8] K. Katahira, K. Watanabe, and M. Okada, in *Journal of Physics: Conference Series*, Vol. 95 (IOP Publishing, 2008) p. 012015.
[9] R. P. Feynman and A. R. Hibbs, *Quantum mechanics and path integration* (McGrawsHill, 1965).
[10] M. Suzuki, Progress of Theoretical Physics **56**, 1454 (1976).
[11] M. Takahashi and M. Imada, Journal of the Physical Society of Japan **53**, 963 (1984).
[12] M. Takahashi and M. Imada, Journal of the Physical Society of Japan **53**, 3765 (1984).
[13] I. Sato, K. Kurihara, S. Tanaka, H. Nakagawa, and S. Miyashita, in *in Proceedings of the Twenty-Fifth Conference on Uncertainty in Artificial Intelligence, ser. UAI '09* (AUAI Press, 2009) pp. 479–486.
[14] I. Sato, *Quantum annealing in statistical machine learning*, Ph.D. thesis, The Unversity of Tokyo (2011).
[15] M. Lichman, "UCI machine learning repository," (2013).
[16] D. Dheeru and E. Karra Taniskidou, "UCI machine learning repository," (2017).
[17] Y. LeCun, L. Bottou, Y. Bengio, and P. Haffner, Proceedings of the IEEE **86**, 2278 (1998).
